# Supplementary figures and images for: A variational deep-learning approach to modeling memory T cell dynamics
Source: PLoS Comput Biol. 2025 Jul 24;21(7):e1013242. doi: 10.1371/journal.pcbi.1013242 (PMC12360662; doi:10.1371/journal.pcbi.1013242)

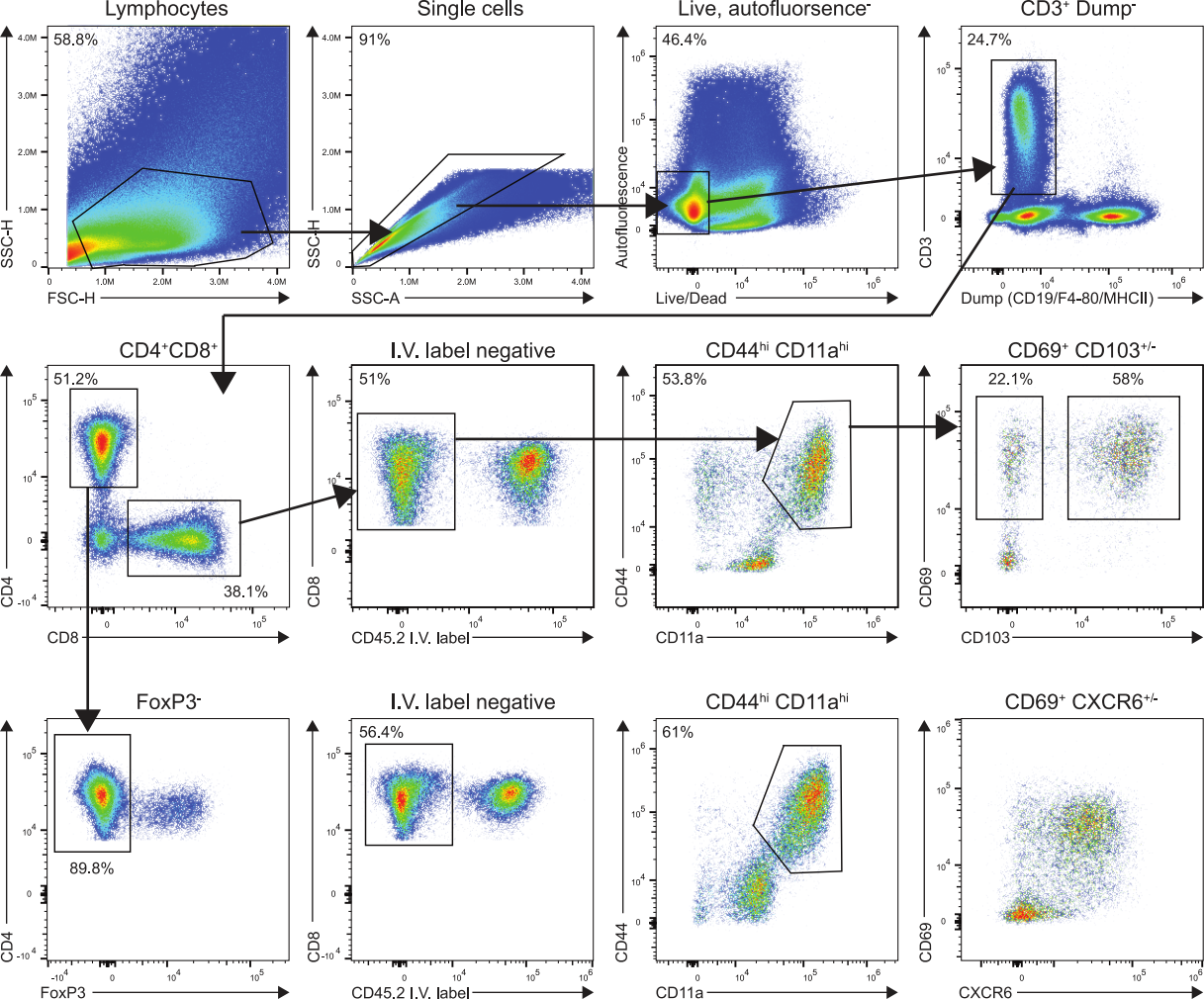

Supplement: S1 Fig — (PDF) [file pcbi.1013242.s002.pdf]

A

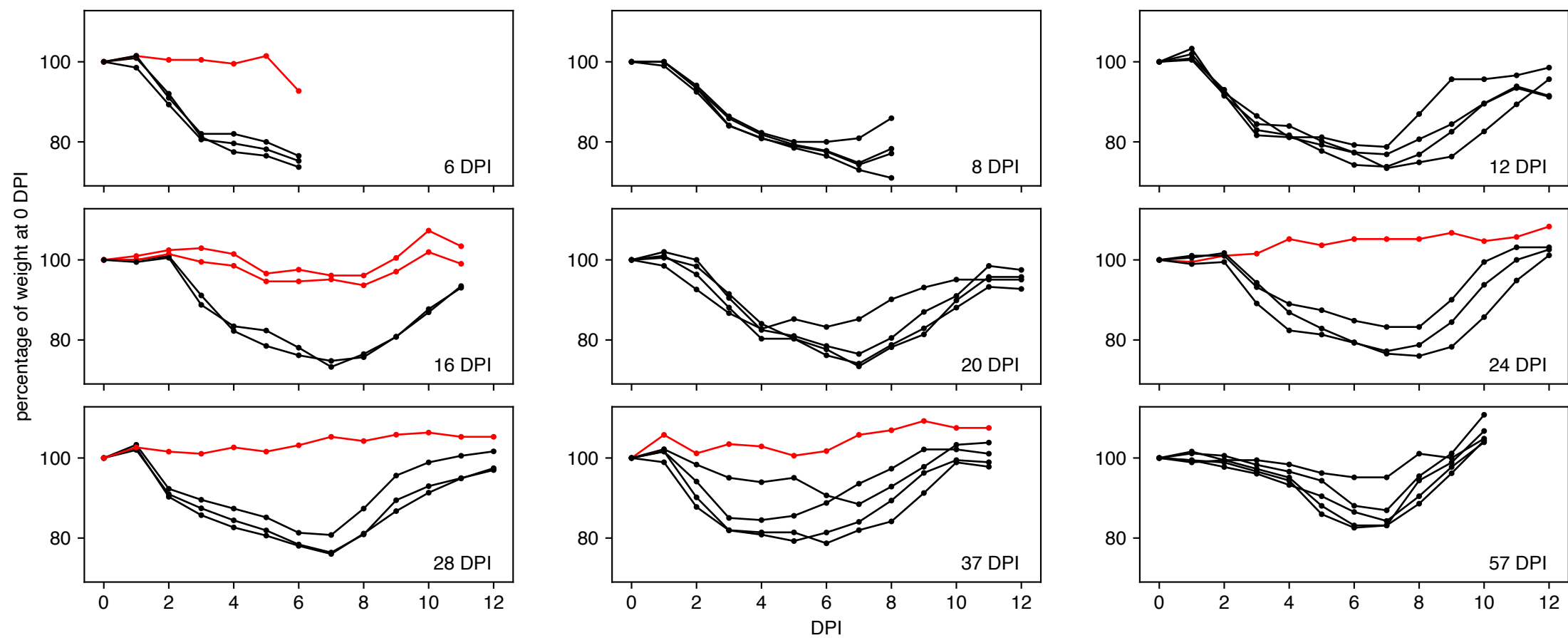

B

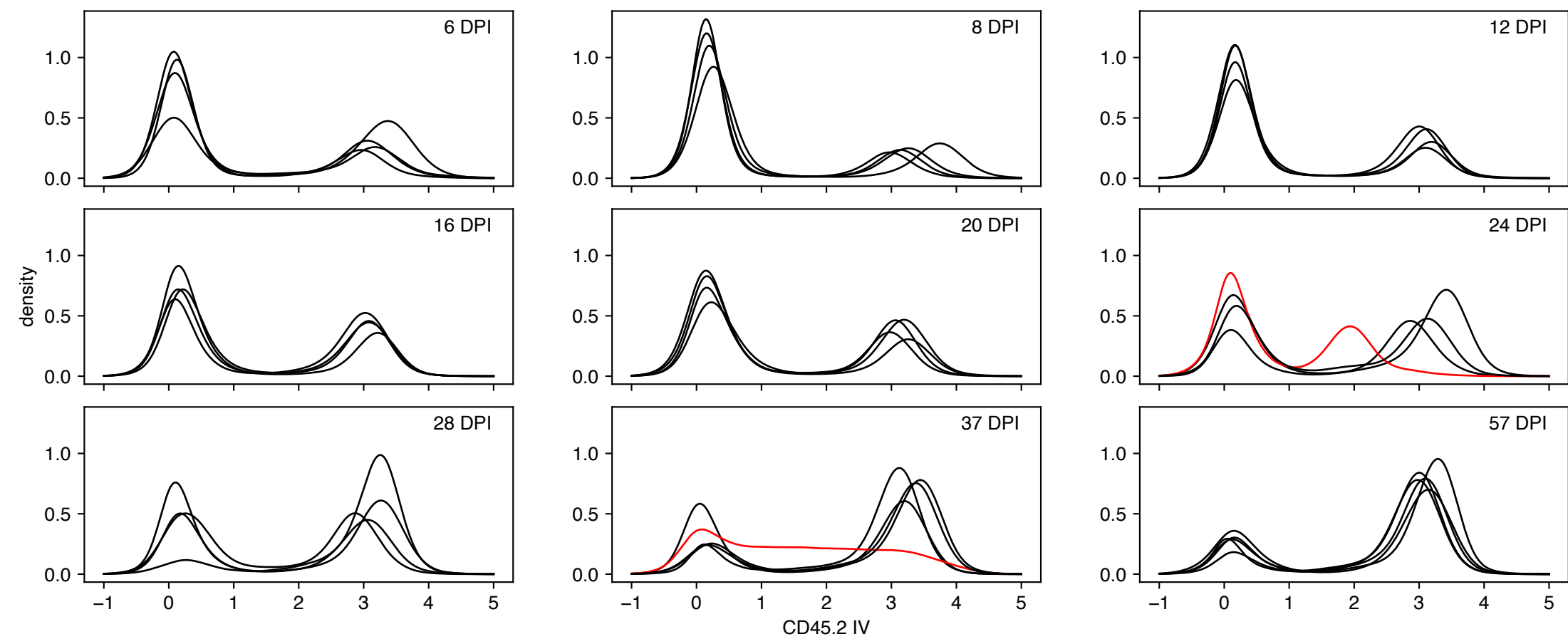

Supplement: S2 Fig — Each cohort contained 4 or 5 mice. In total data from 38 mice is presented. A. The curves indicate the percentage of the weight relative to that on the day of infection. The red curves correspond to mice that were excluded from further analysis due to lack of weight loss. B. Distribution of the I.V. label (CD45.2 IV) for each mouse. The red curves correspond to mice that were excluded from further analysis due to poor I.V. labeling. (PDF) [file pcbi.1013242.s003.pdf]

A

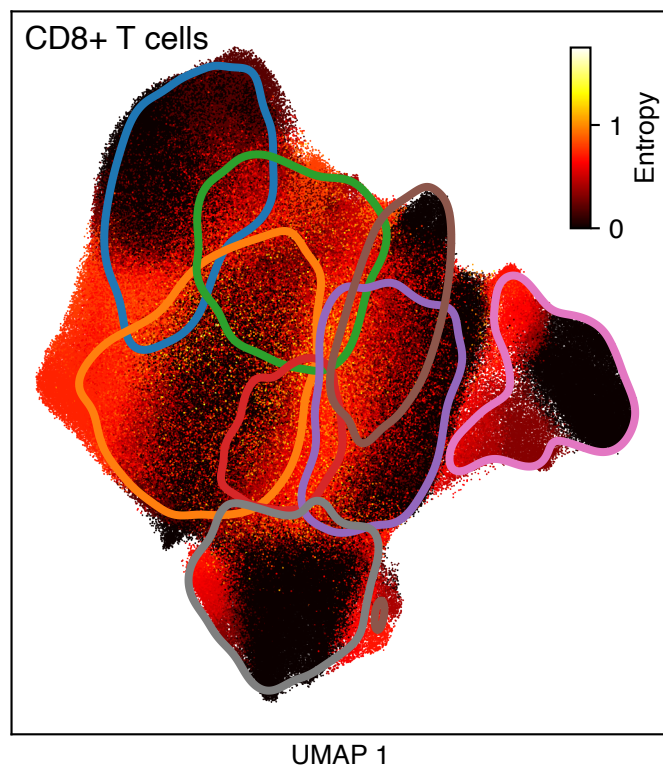

B

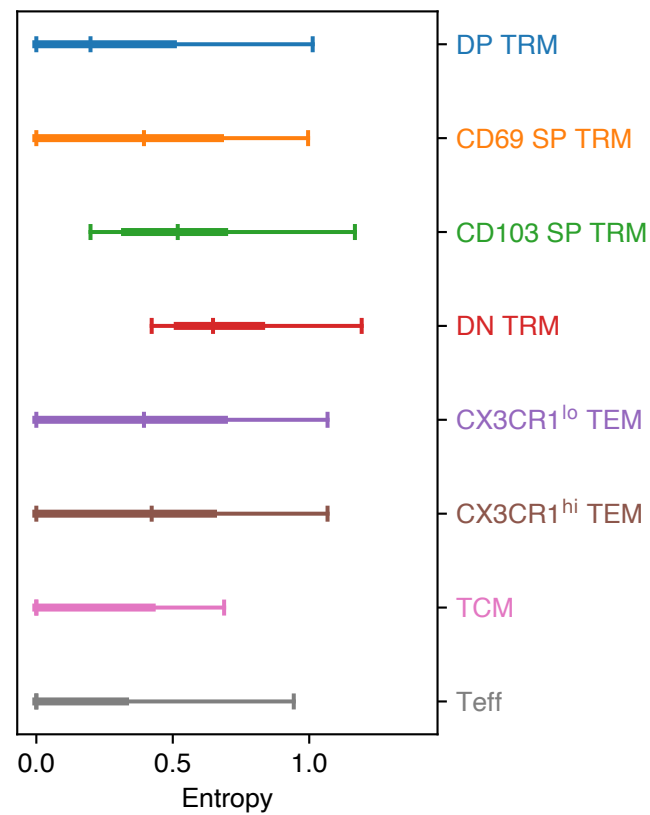

C

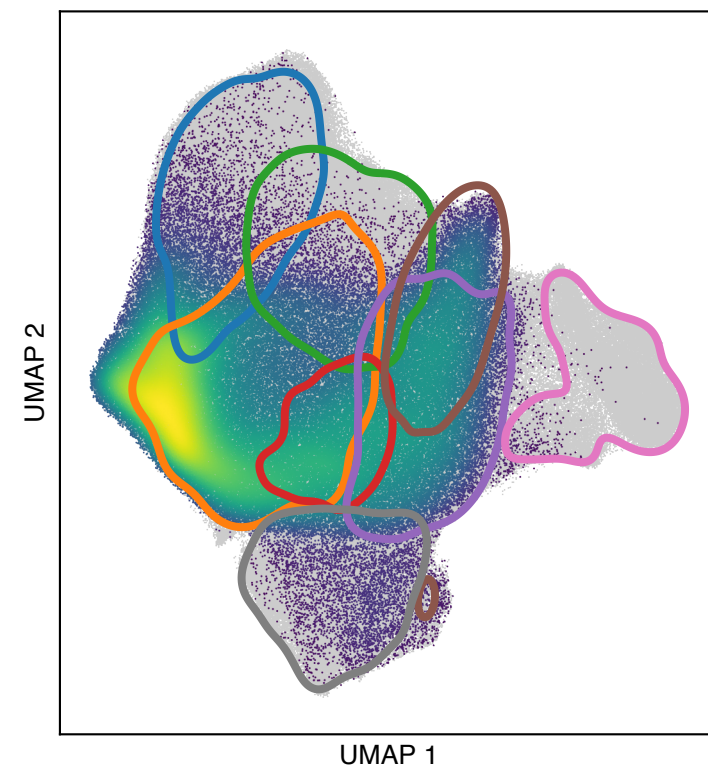

D

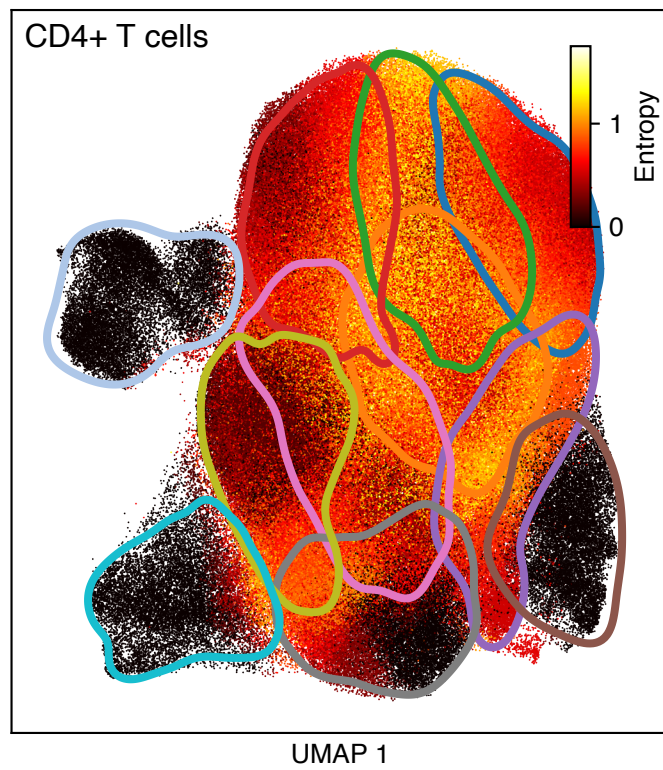

E

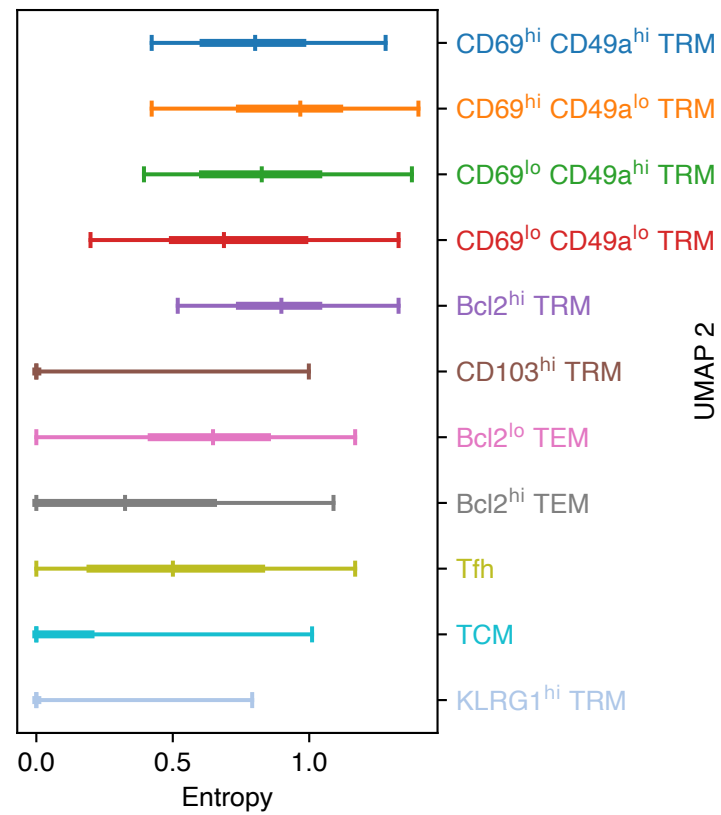

F

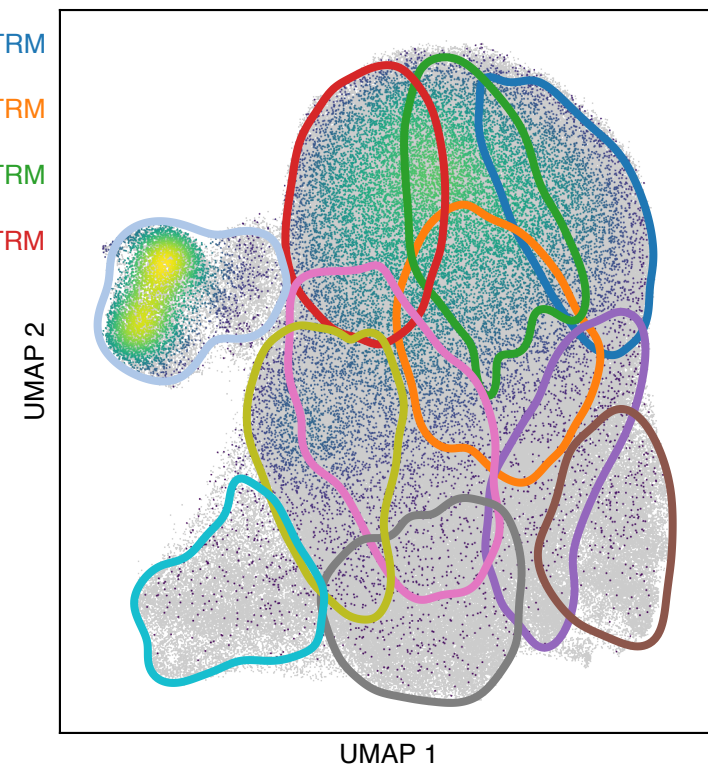

Supplement: S3 Fig — Results are based on data from n = 27 mice. A. Entropy of CD8 T cell population assignment based on 20 Leiden clustering runs with different random seeds. Black dots corresponds to very certain assignments, yellow dots to highly uncertain assignments. The colored contours indicate the location of the different subpopulations in the UMAP. B. Entropy distribution per cluster. The bar plots show the median, IQR and 2.5–97.5 percentile range. The color of the bars and labels correspond to the contours in panel A. C. Distribution of IAV NP-specific CD8 T cells in the UMAP with contours indicating clusters. Gray dots indicate bulk antigen-experienced cells. D–F. Same as panels A–C, but for CD4 T cell data. (PDF) [file pcbi.1013242.s004.pdf]

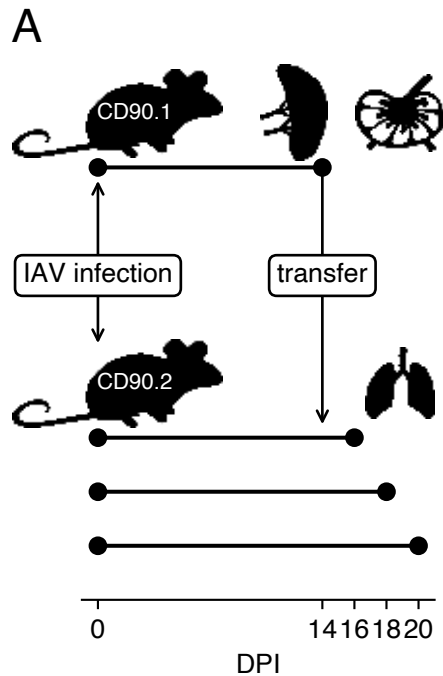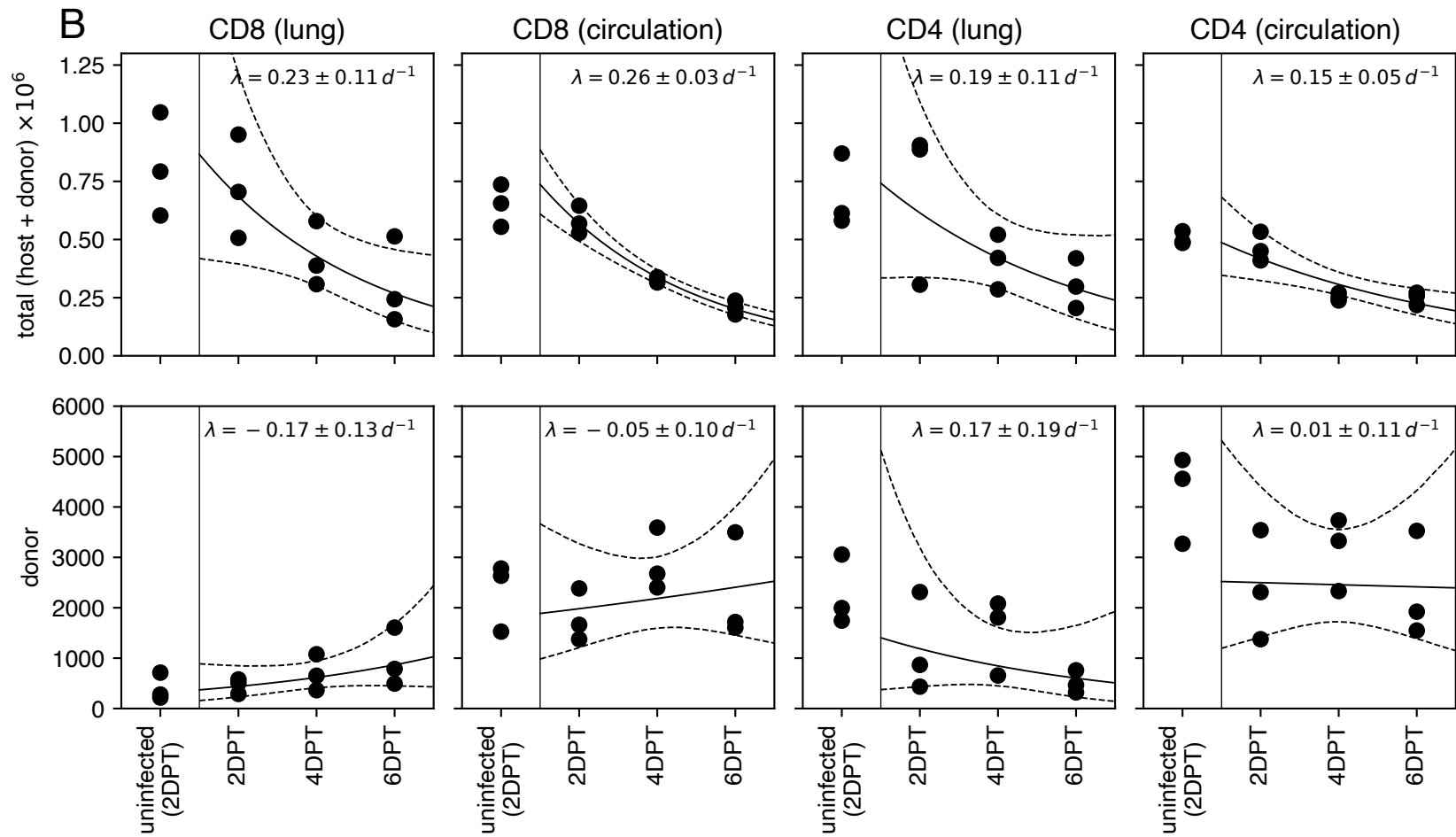

Supplement: S5 Fig — Results are based on data from n = 12 mice in total, with 3 mice per group. A. Design of the congenic transfer experiment. B. Numbers of protected and labeled, antigen-experienced CD8 and CD4 T cells from host and donors combined (upper panels) and donors only (lower panels). We fitted a log-linear model to the cell counts (solid line: ML estimate, dashed lines: 95% confidence envelope). The indicated λ is the estimated net loss rate (± standard error). (PDF) [file pcbi.1013242.s006.pdf]

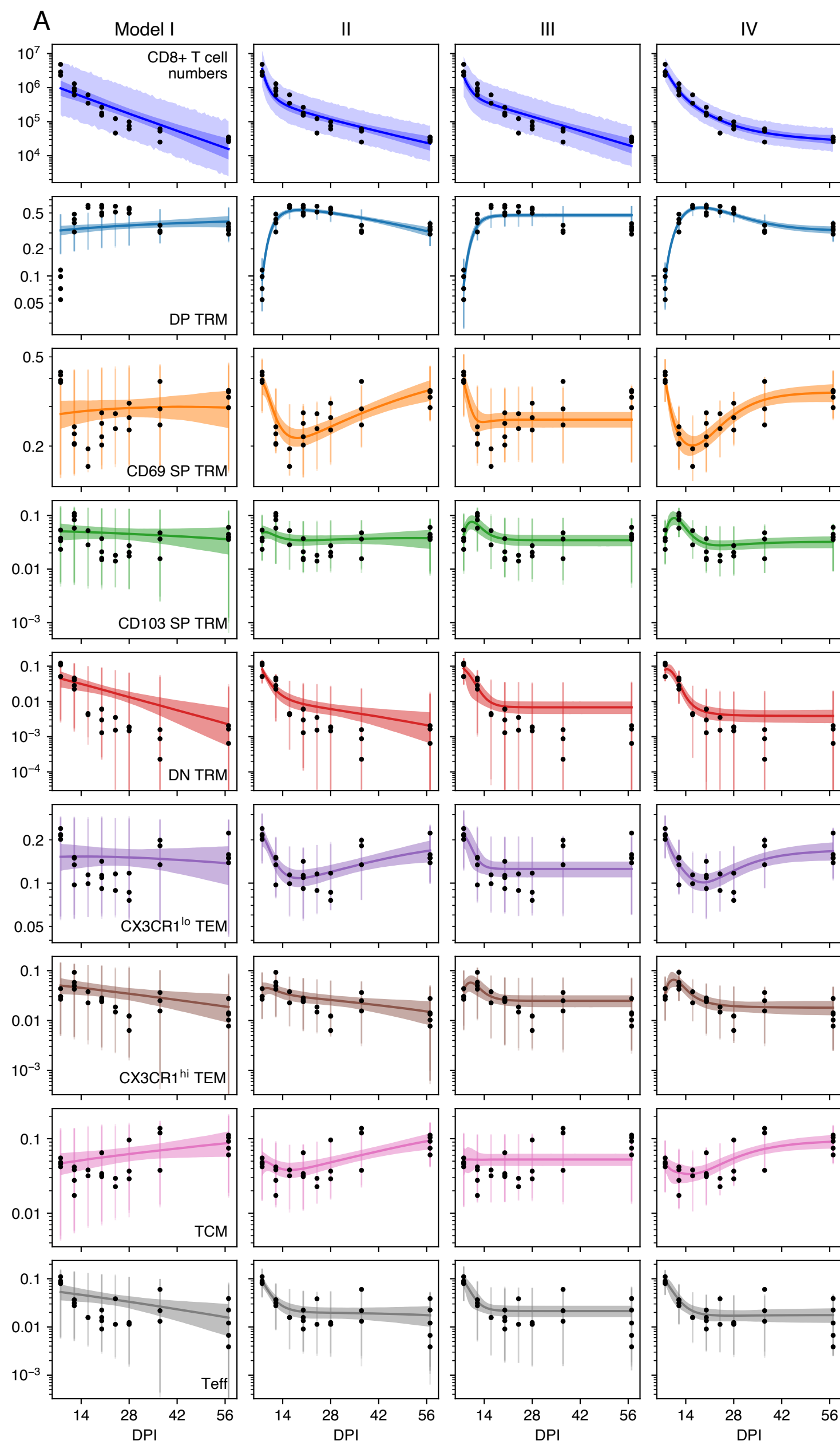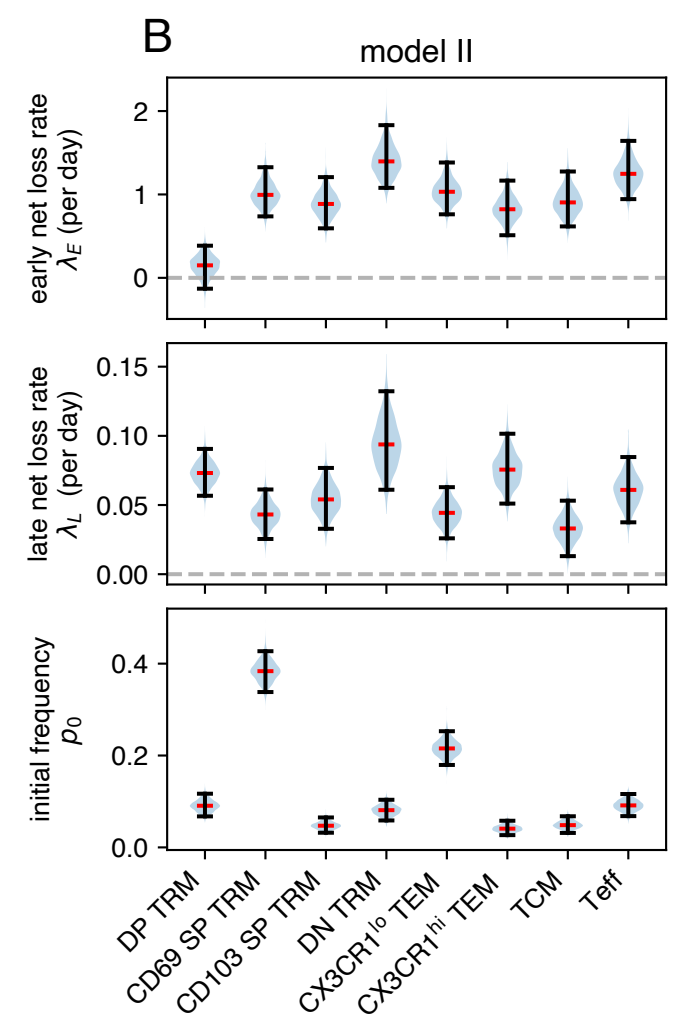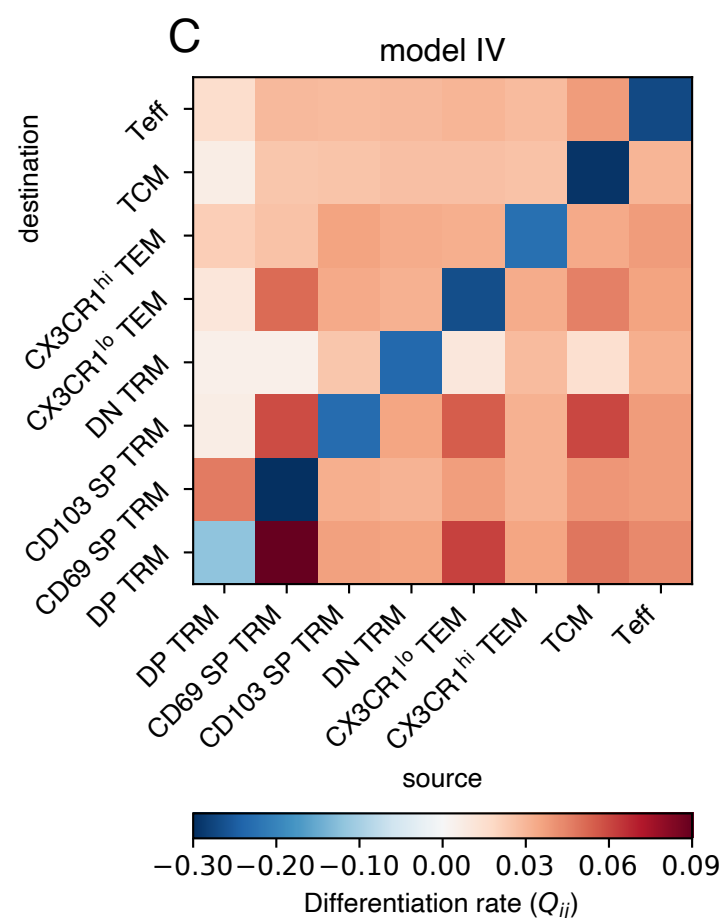

Supplement: S6 Fig — Results are based on data from n = 27 mice. A. Top panels: numbers of CD8+ T cells in the lung (black dots) and the model fits (blue lines). Remaining panels: observed and predicted subpopulation frequencies. Bands indicate 95% credible envelopes, bars indicate the 2.5 and 97.5 percentiles of the posterior predictive distributions. B. For model II, marginal posterior distributions of the parameters as violin plots (blue), with median (red) and 95% credible intervals (CrI; black). C. Differentiation rates in model IV. Diagonal; total egress rates by differentiation are represented by negative values. (PDF) [file pcbi.1013242.s007.pdf]

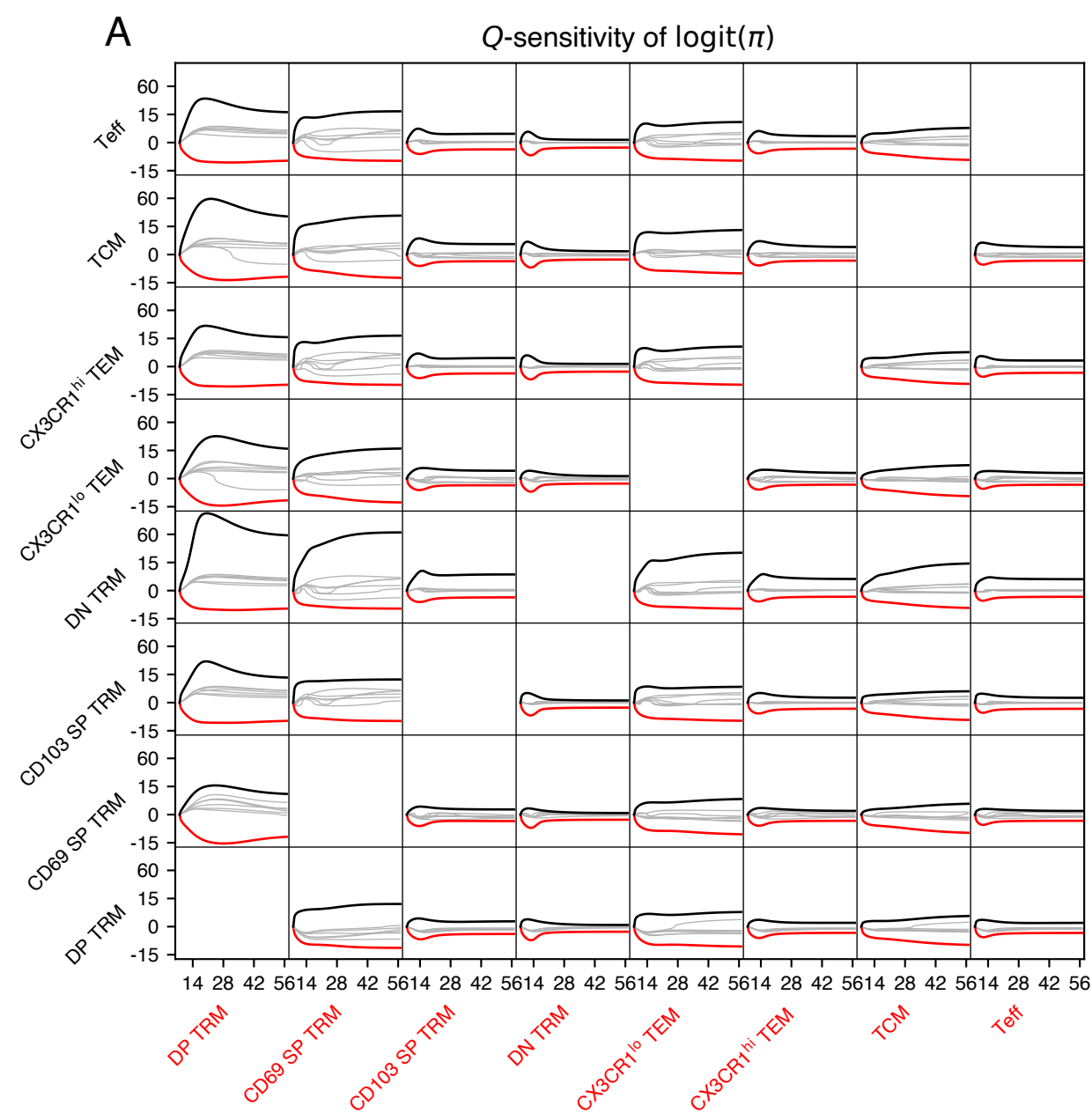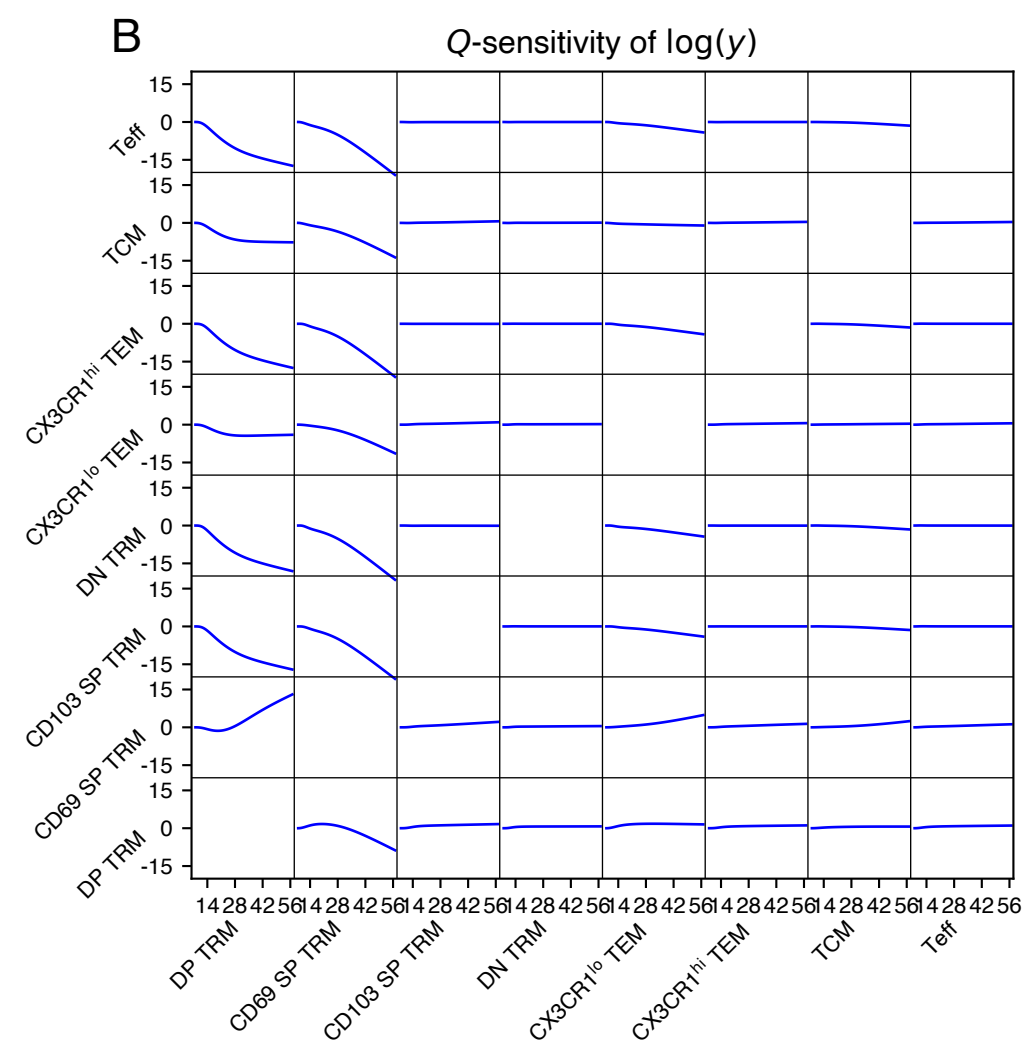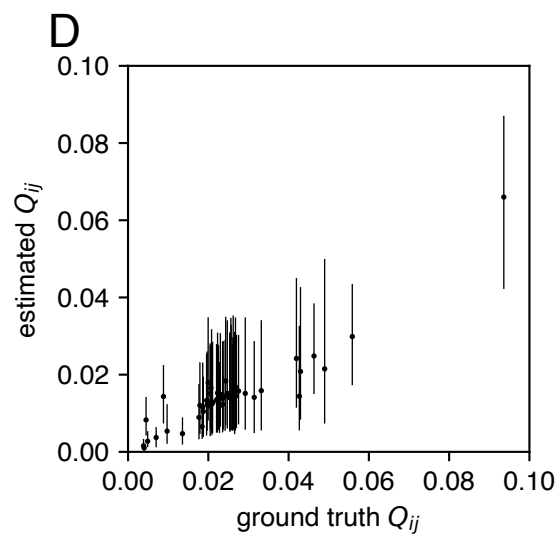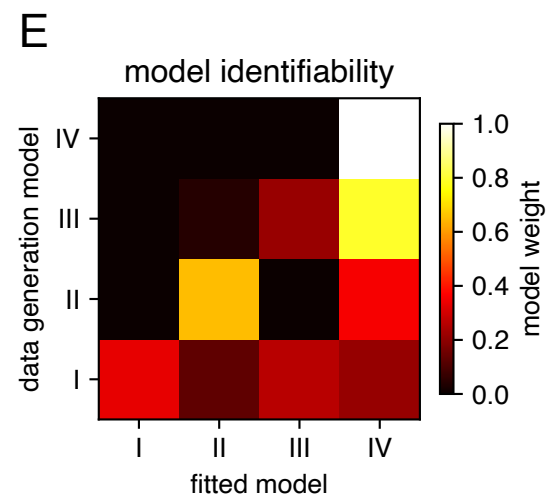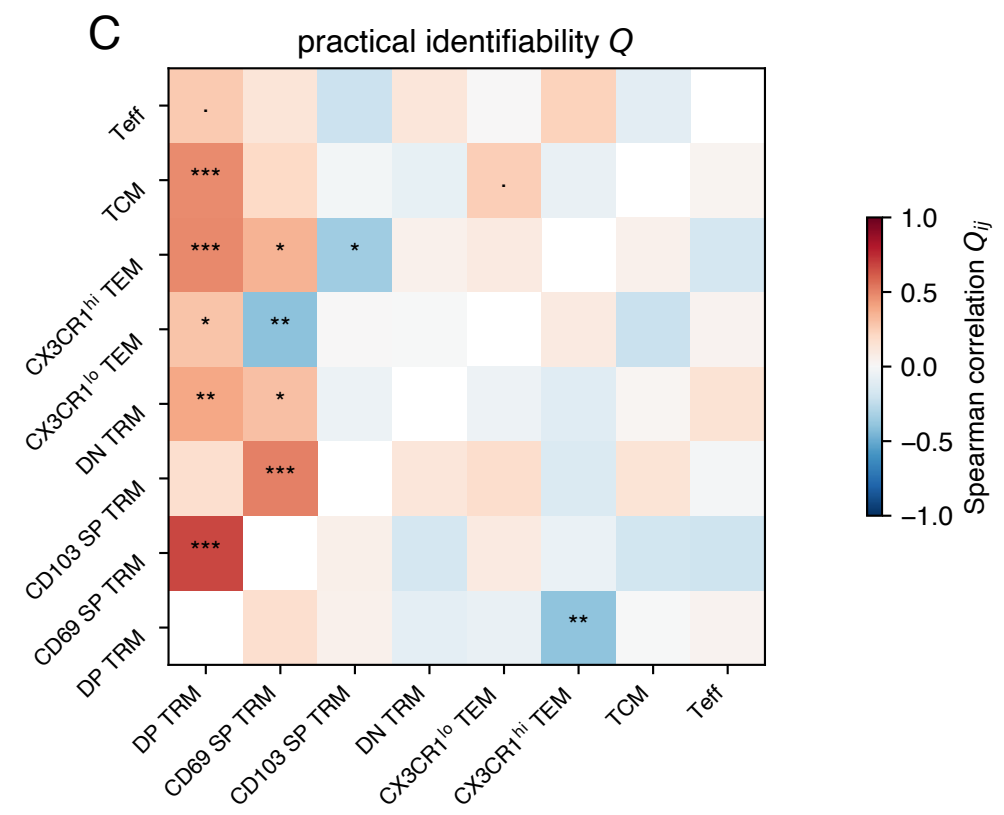

Supplement: S7 Fig — A. Sensitivity of relative cluster sizes (πk(t)) with respect to differentiation rates Qij. Each plot contains d = 8 curves ∂logit(πk(t))/∂Qij. The primary effects are highlighted in black (i = k) and red (j = k), while the secondary effects are shown in gray. The y-axes are shown on a square-root-scale. B. Sensitivity of total population size (Y(t)) with respect to differentiation rates Qij. The curves correspond to ∂log(Y(t))/∂Qij. C. Practical identifiability scores of differentiation rates Qij. The heatmap shows the correlation coefficient between the ground truth value of Qij, and the estimated value. The stars indicate the levels of statistical significance (· p < 0.1, *p < 0.05, **p < 0.01, ***p < 0.001) based on a range of 51 ground truth Qij values. D. A single pseudo-dataset is simulated with model IV, using parameters estimated from the true data. Model IV is then fit to the pseudo-data, and for each pair of populations (i,j) we show the estimate versus the ground truth value of Qij. E. Model identifiability. Data was simulated with and fit to each of the four models, resulting in 16 model fits. For each simulated dataset, the four model fits are compared with model weights (shown in color). High diagonal model weights indicate that the ground-truth model is correctly identified. Shown is the median of 3 simulations for each model. (PDF) [file pcbi.1013242.s008.pdf]

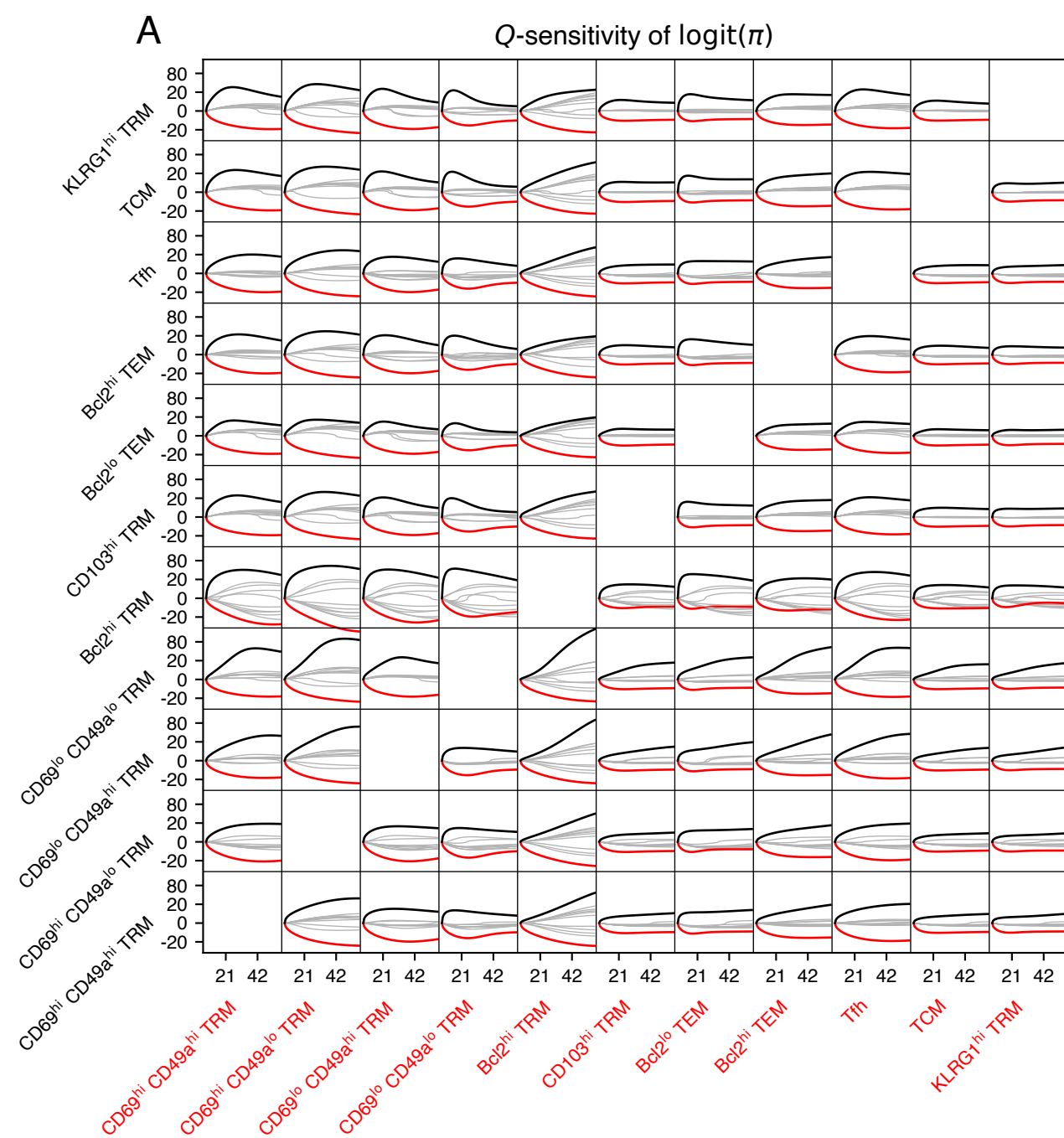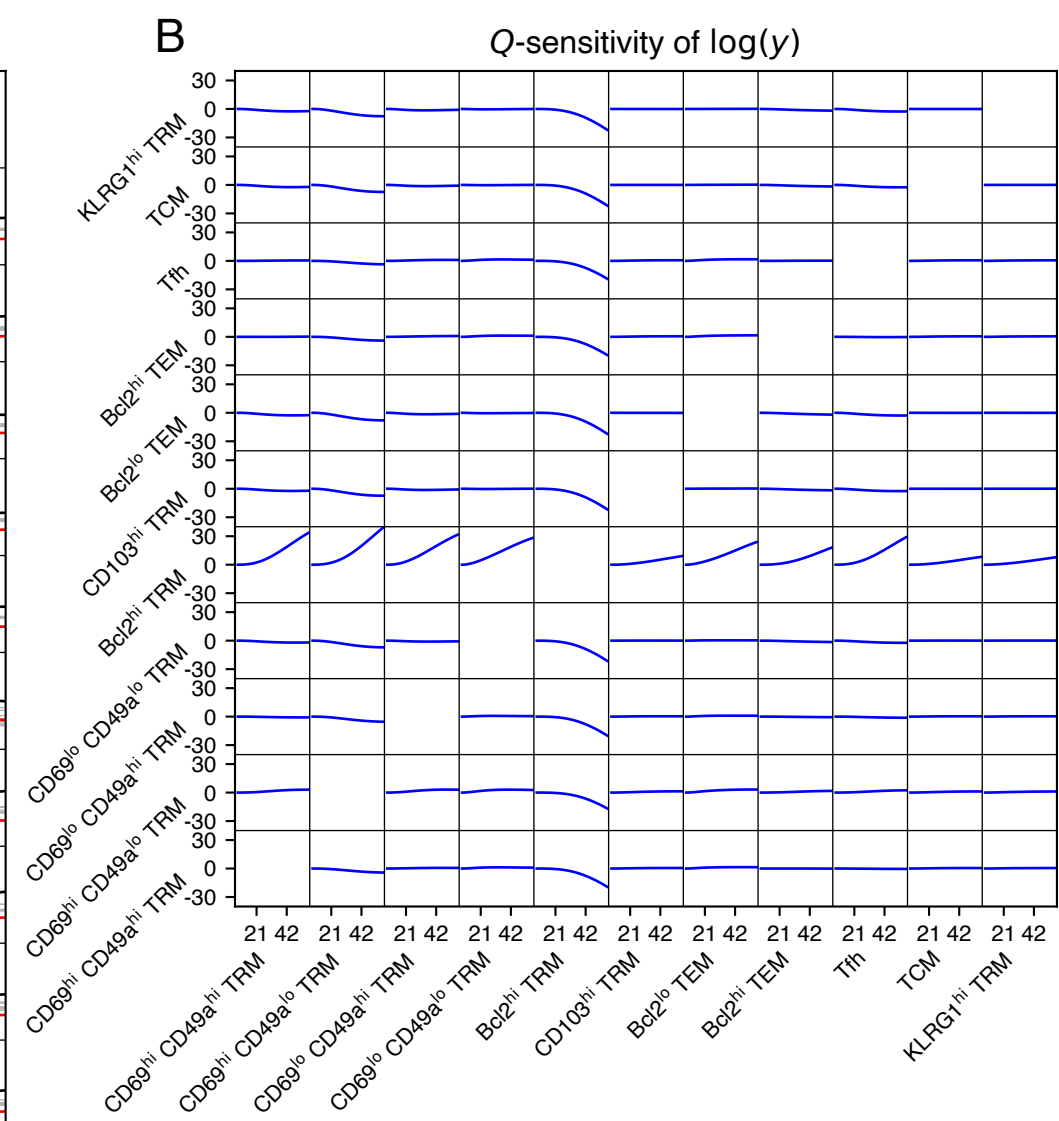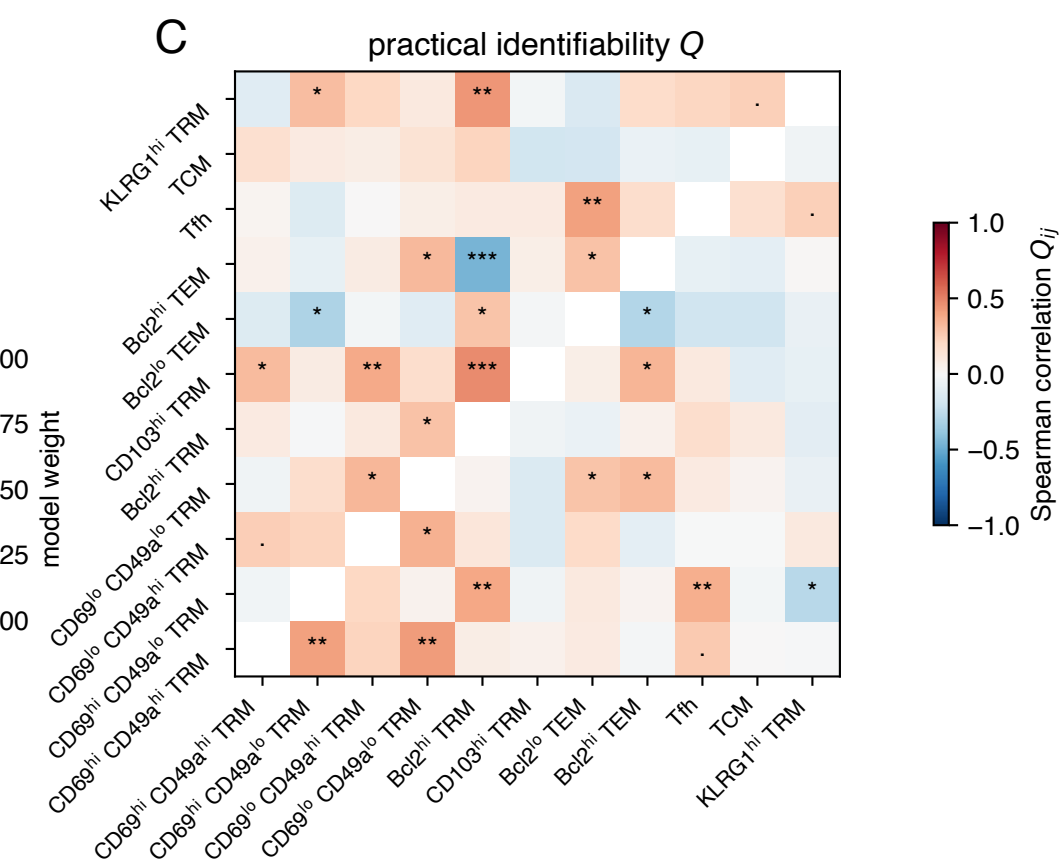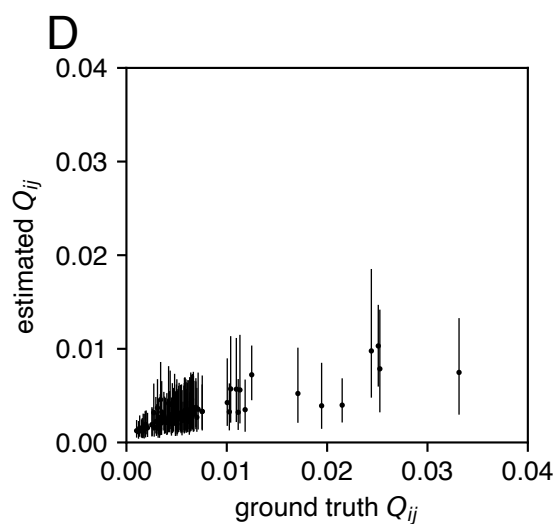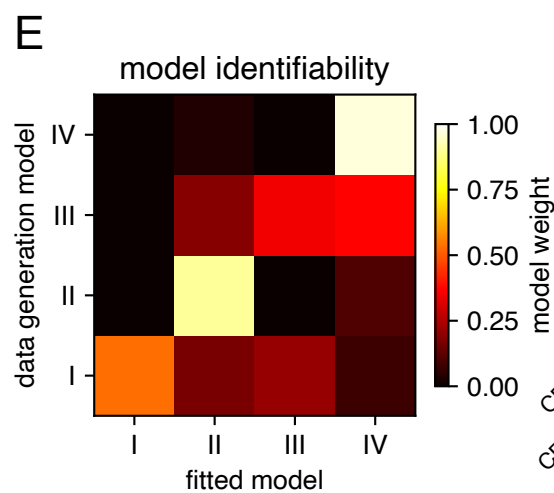

Supplement: S8 Fig — See the caption of S7 Fig for details. In this case we have d = 11 populations, and for panel D we simulated and fitted with model III. (PDF) [file pcbi.1013242.s009.pdf]

A

CD8 lineage

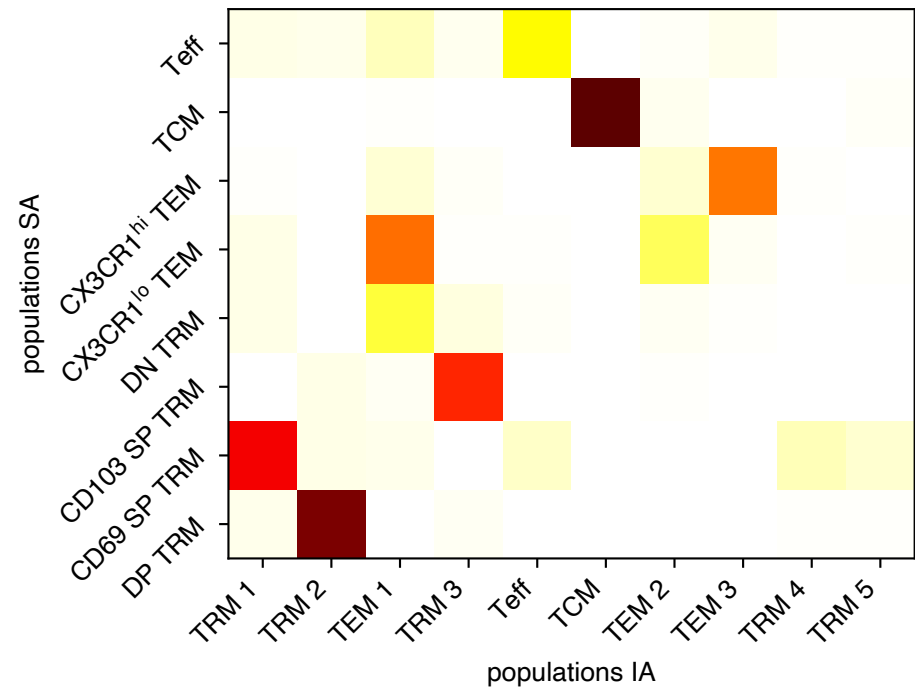

B

CD4 lineage

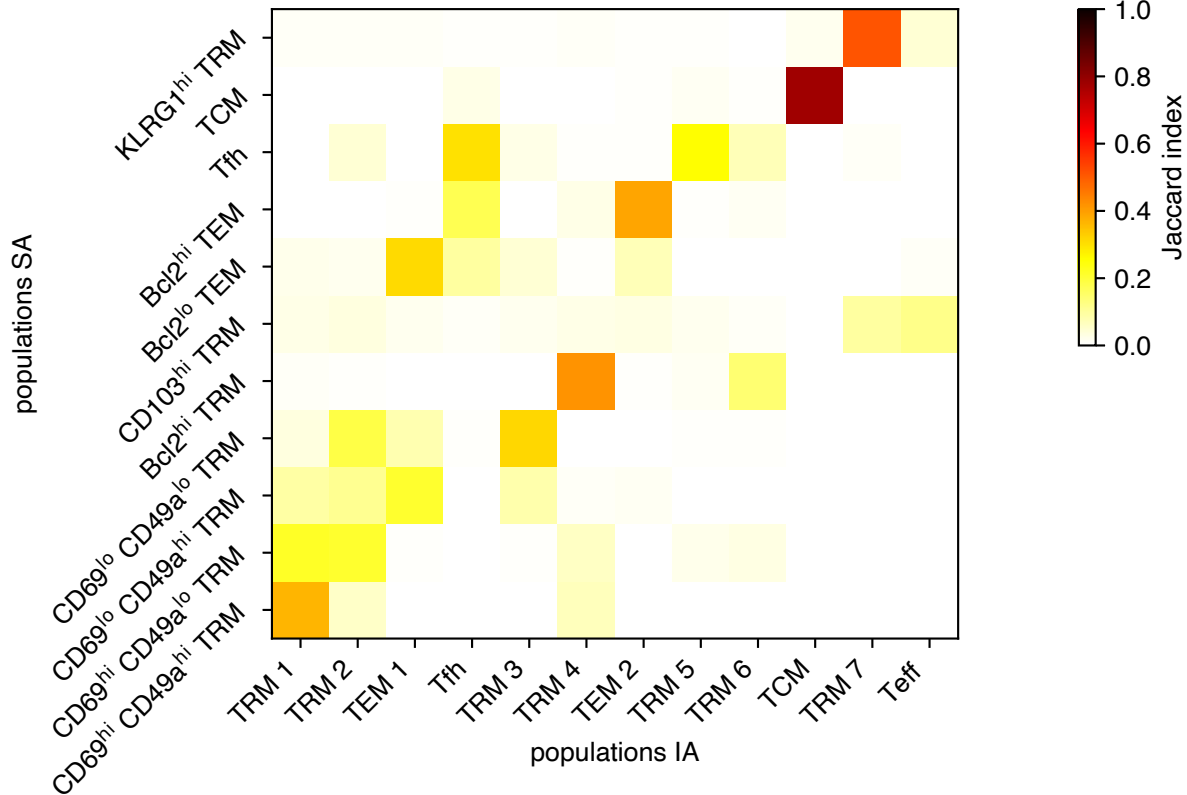

Supplement: S9 Fig — Results are based on data from n = 27 mice. The sequential (SA) and integrated (IA) approaches both assign each cell to a subpopulation, but for any given cell this assignment may differ between the approaches. To quantify the similarity, we calculated the Jaccard index for each pair of subpopulations (one from the IA and the other from the SA). The Jaccard index is ratio of the number of cells that are in both clusters, and the number of cells that are in either one of the clusters. A. Results for CD8 T cell data. B. Results for CD4 T cell data. (PDF) [file pcbi.1013242.s010.pdf]

**A**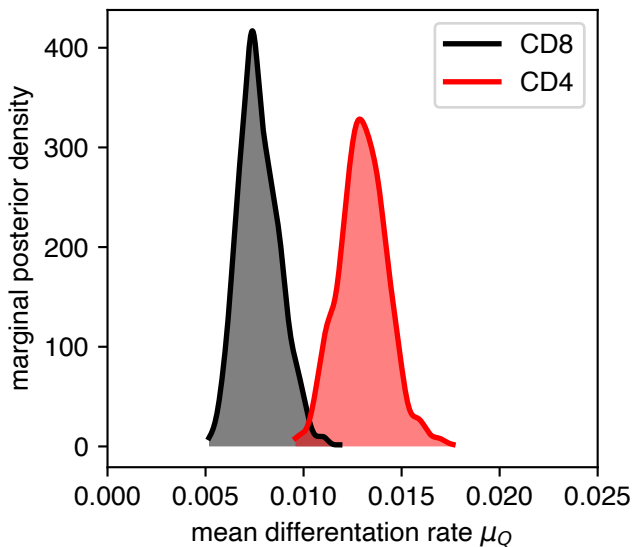**B**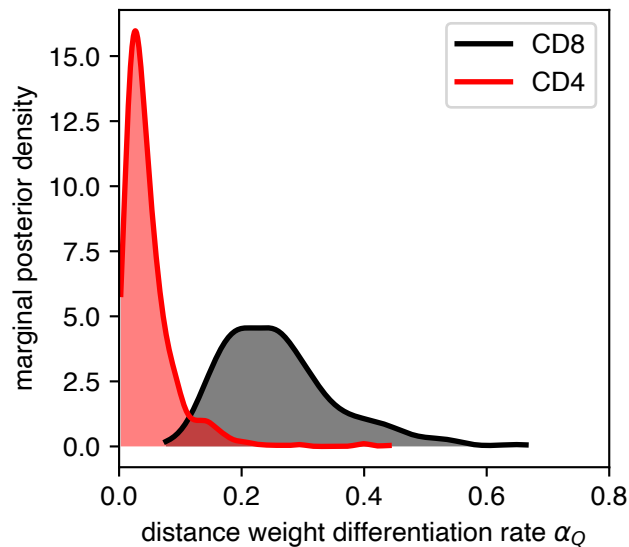**C**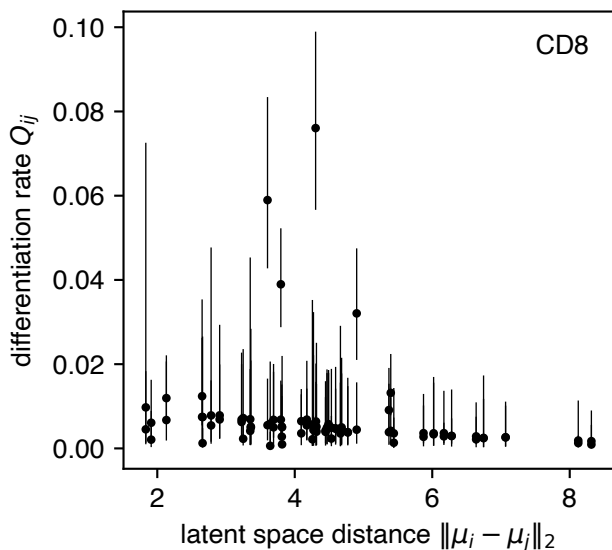**D**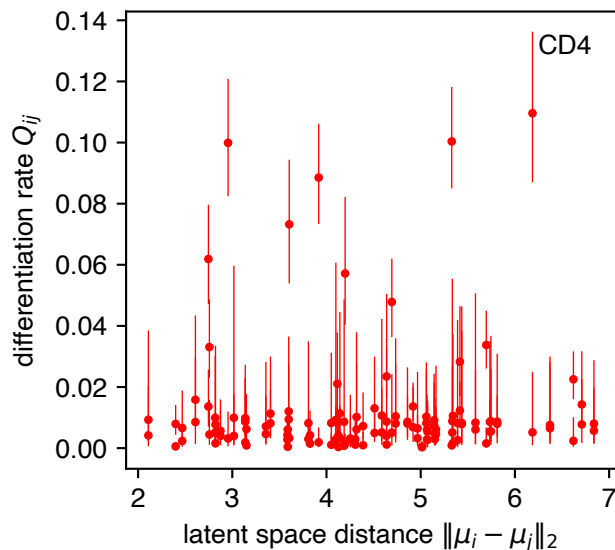

Supplement: S10 Fig — A. Marginal posterior density of the mean differentiation rate μQ. B. Marginal posterior density of the weight αQ of the distance matrix Dij=‖μi−μj‖2 on the differentiation matrix elements Qij. C. and D. 95% credible intervals (lines) and posterior medians (dots) of Qij as a function of the distance between the mixture components i and j in the latent space. (PDF) [file pcbi.1013242.s011.pdf]

**A**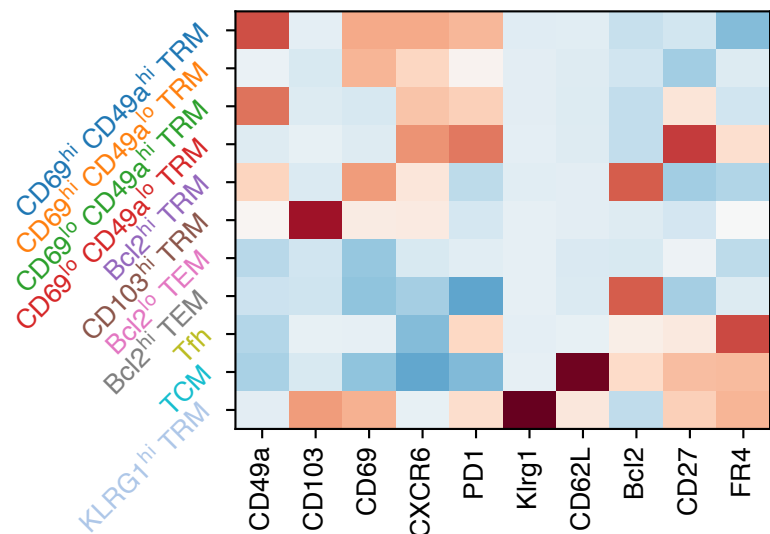**B**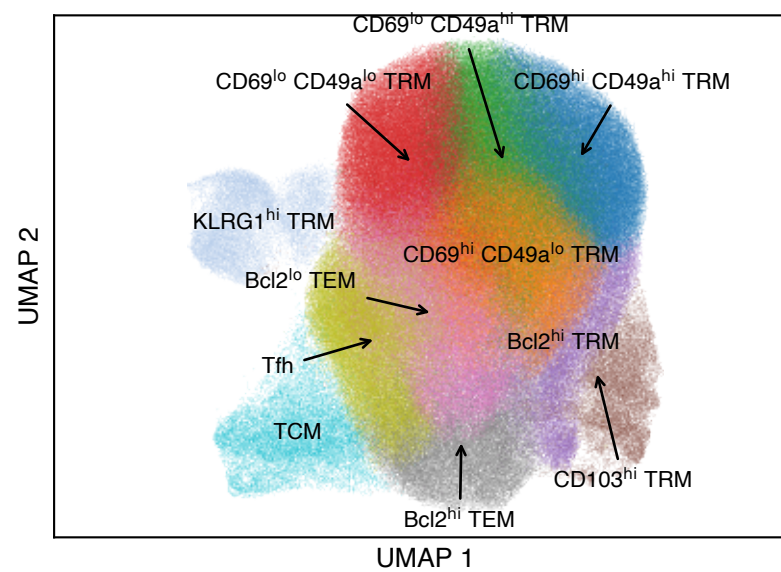**C**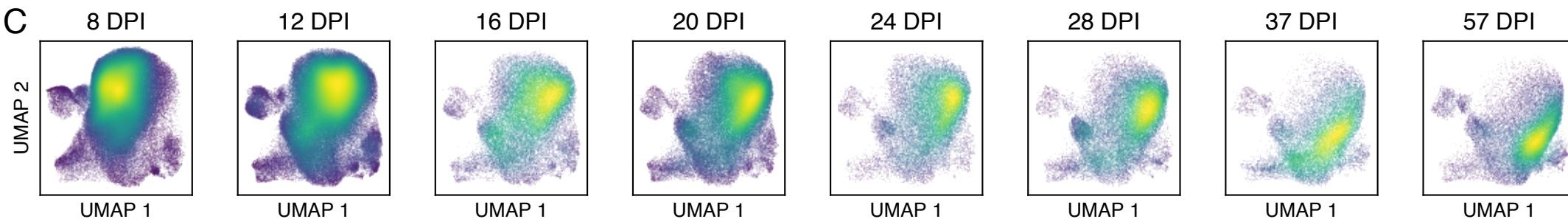**D**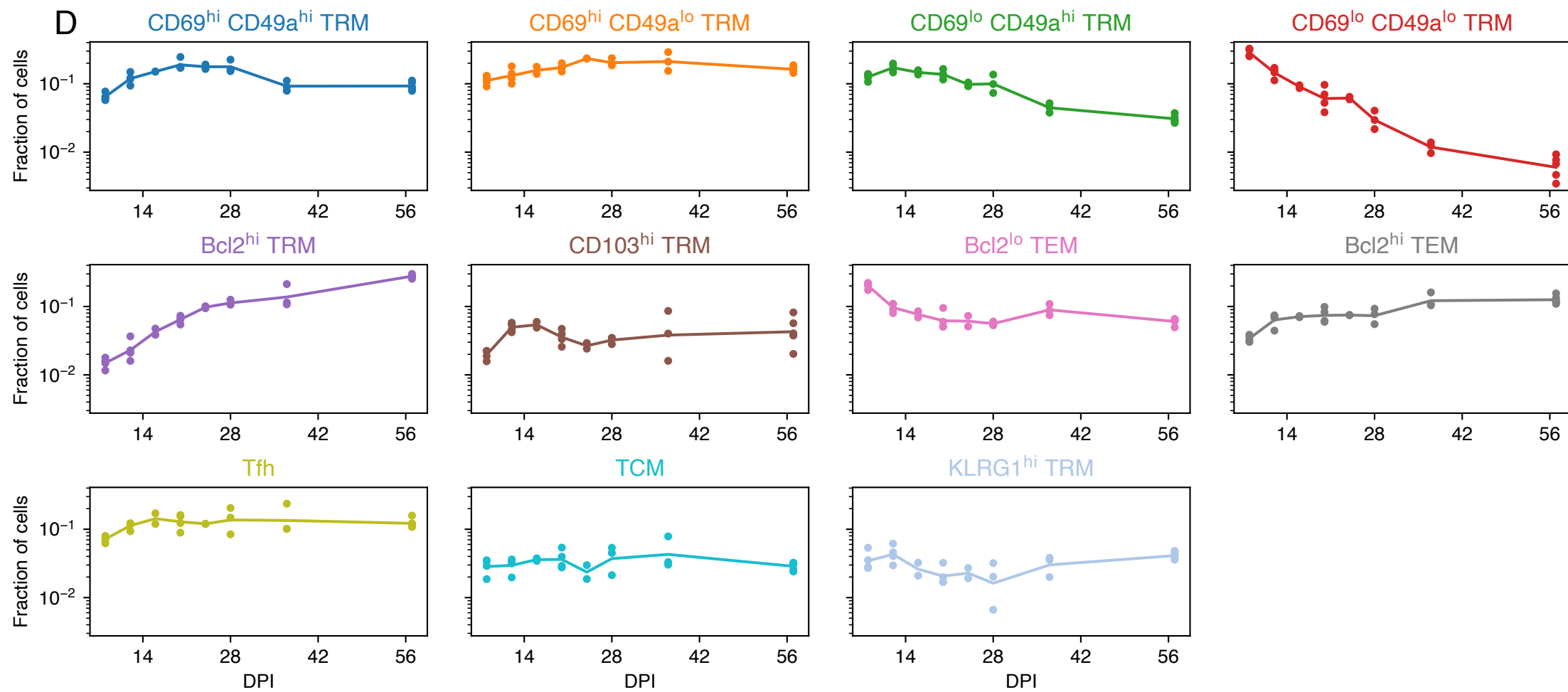

Supplement: S11 Fig — Results are based on data from n = 27 mice. A. Marker expression heatmap for selected markers and consensus T-cell populations. B. UMAP of the marker expression data, colored by annotation. C. UMAPs of marker expression data, split by day post infection (DPI). The color scale reflects cell density in UMAP space. D. Time series of the fraction of cells in each cluster. The lines show a linear interpolation on the log scale. (PDF) [file pcbi.1013242.s012.pdf]

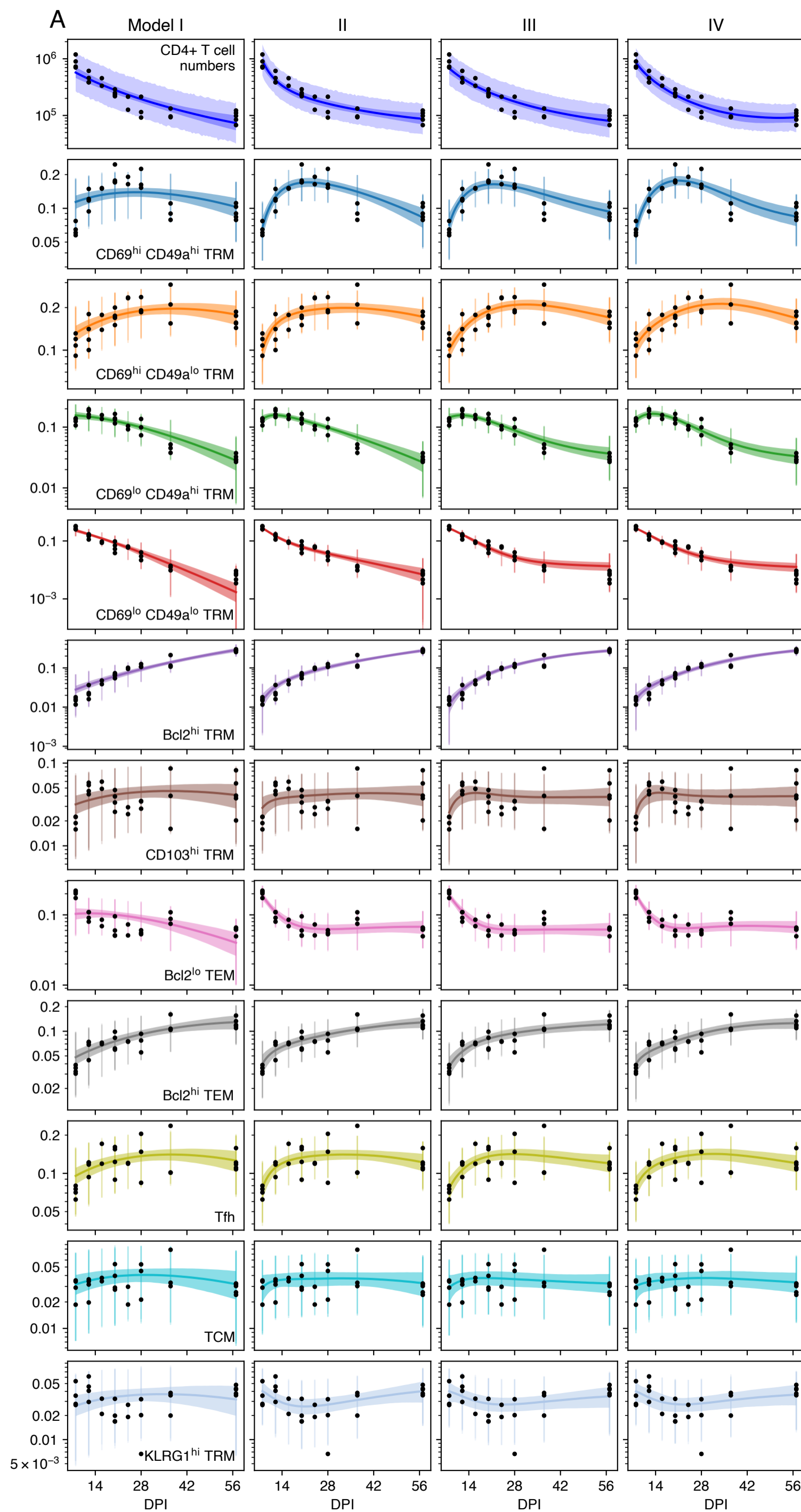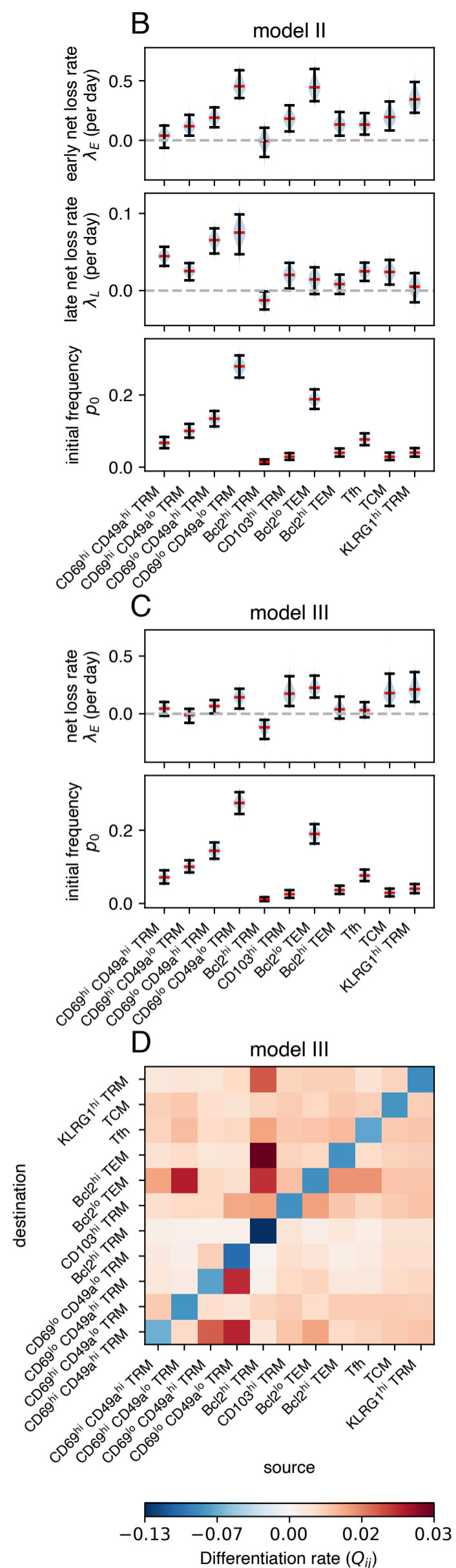

Supplement: S12 Fig — Results are based on data from n = 27 mice. A. Data and predictions from fitted models. Top panels show total antigen-experienced CD4 T cell counts in the lung, other panels show relative population sizes of each of the subpopulations. B. Parameter estimates using model II. C. Parameter estimates using model III. D. Estimated differentiation matrix in model III. (PDF) [file pcbi.1013242.s013.pdf]

**A**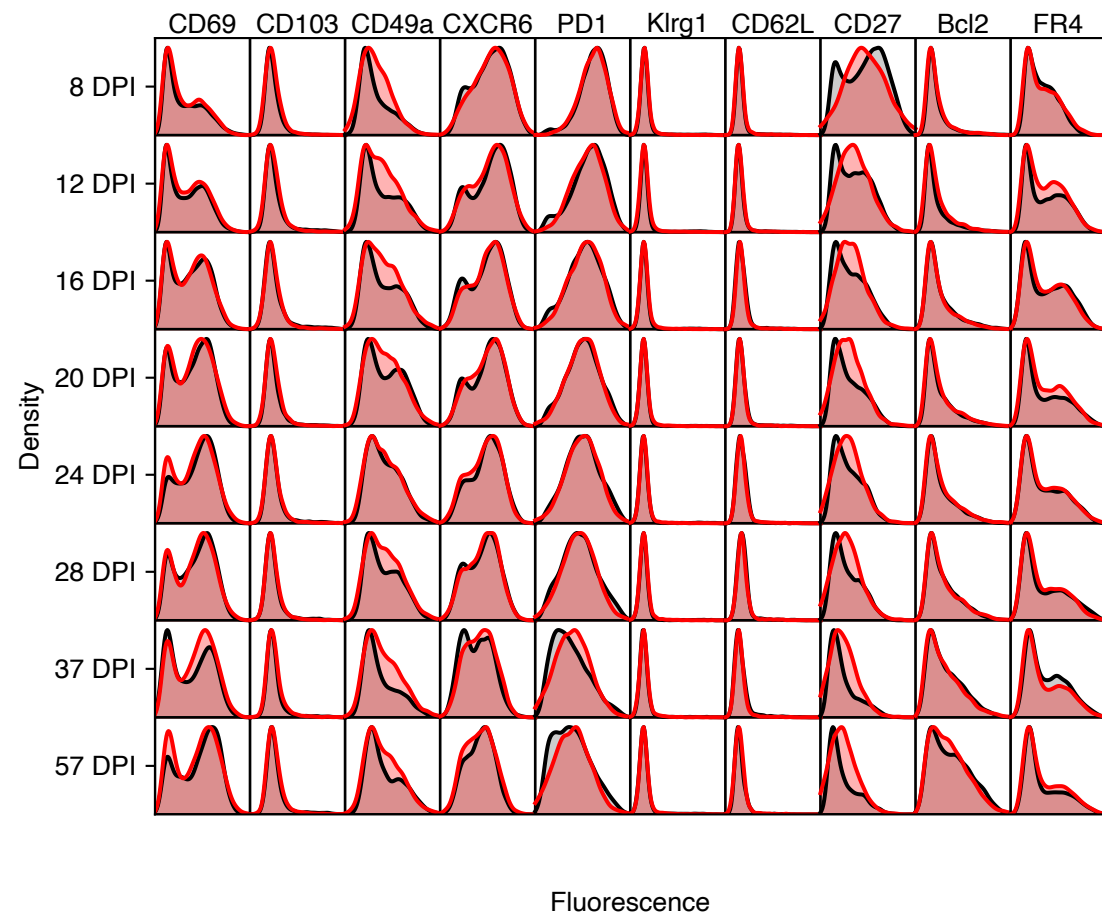**B**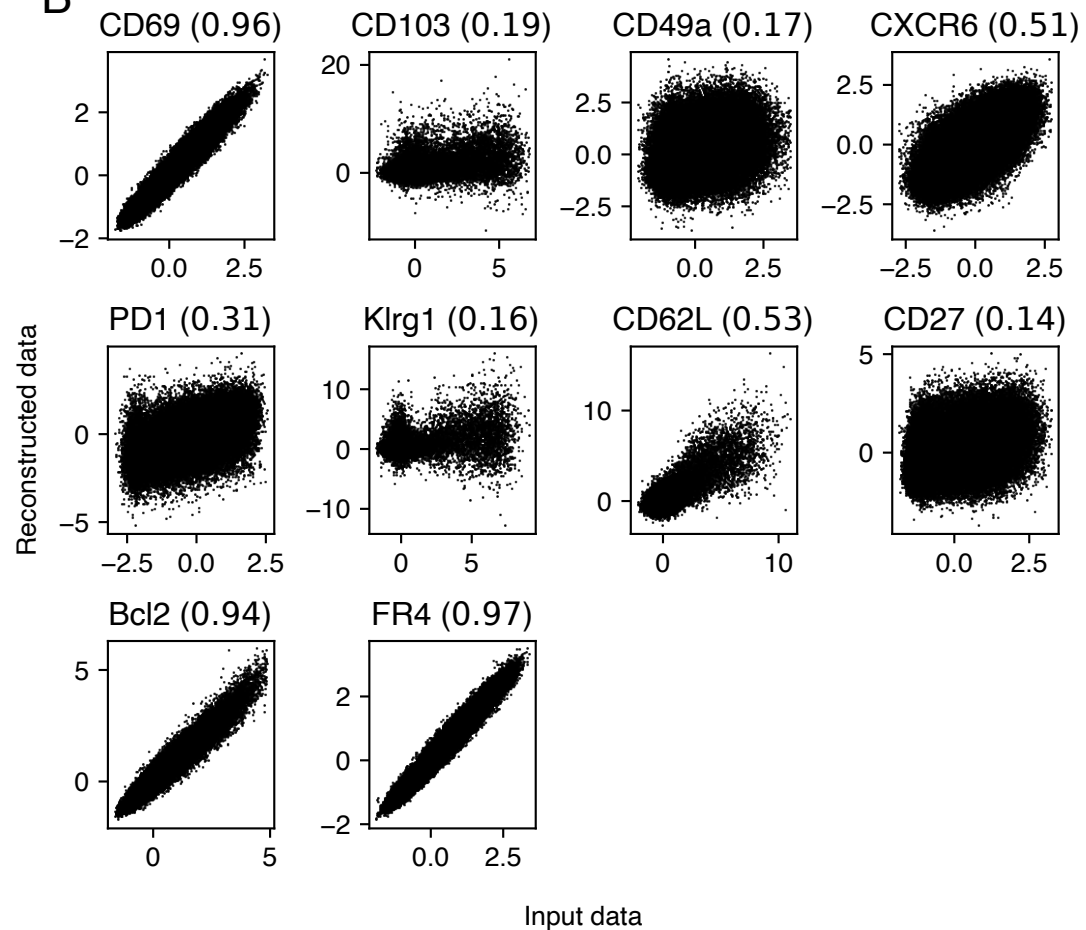

Supplement: S13 Fig — Results are based on data from n = 27 mice. A. Marginal distributions of marker expression (cf. Fig 1, panel I). Data is shown in black, simulated data is shown in red. B. Input data and reconstruction using the autoencoder model. The number in brackets is the coefficient of determination (R2). (PDF) [file pcbi.1013242.s014.pdf]

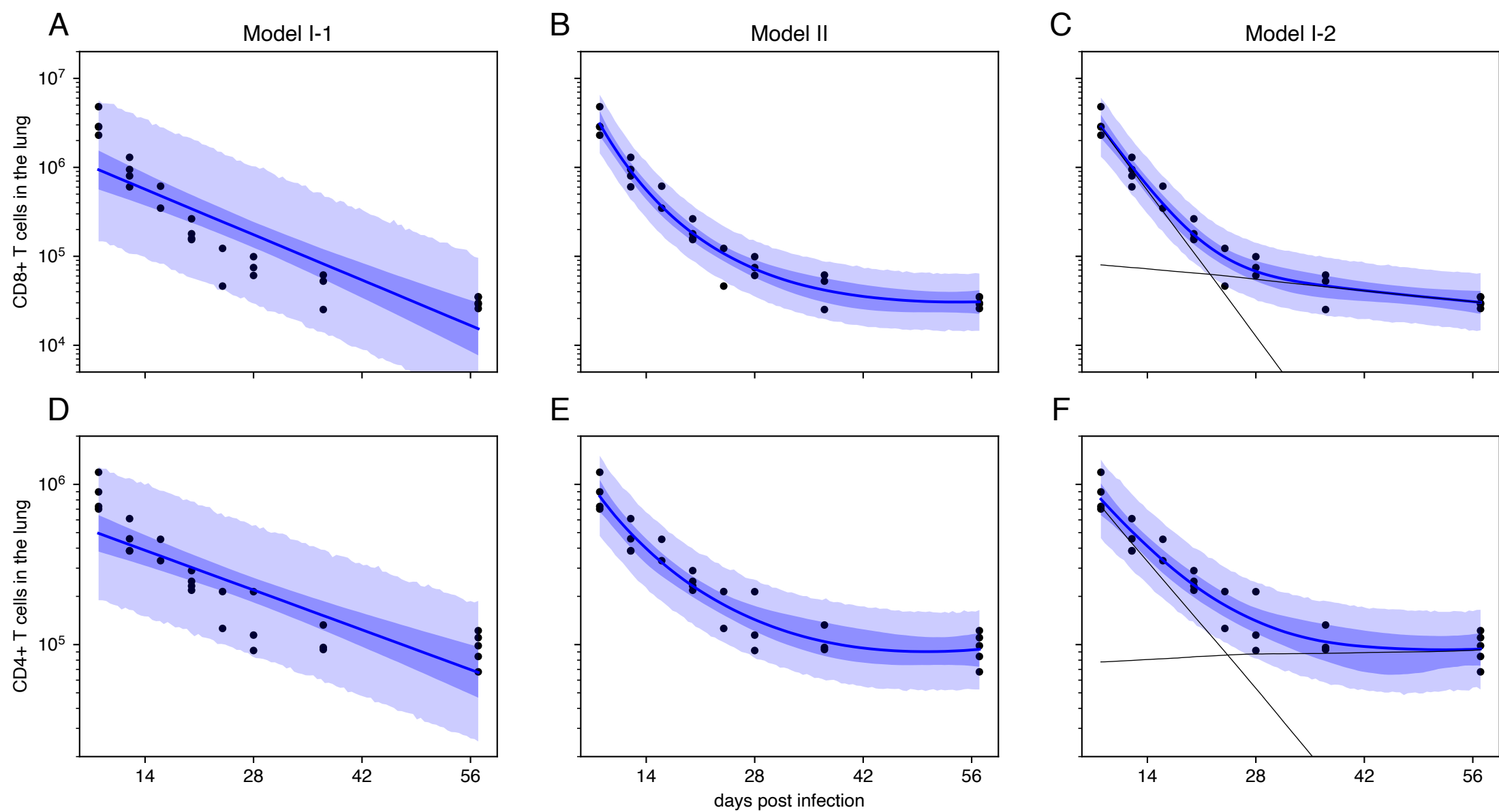

Supplement: S14 Fig — Results are based on data from n = 27 mice. A. time-homogeneous model with a single compartment (i.e. a log-linear model) fit to CD8 T-cell count data (dots). The model fit is shown as a blue curve (posterior median), with 95% CrI as a dark-blue band. The light-blue band shows the posterior predictive interval (i.e. simulated observations). B. Fit of model with a single compartment, but with time-dependent net loss rates λ(t). C. Fit of a time-homogeneous model with two compartments. The population sizes (posterior median) of the two populations are shown as black curves. D–F. Fits of the three models to CD4 T cell counts. (PDF) [file pcbi.1013242.s015.pdf]

A

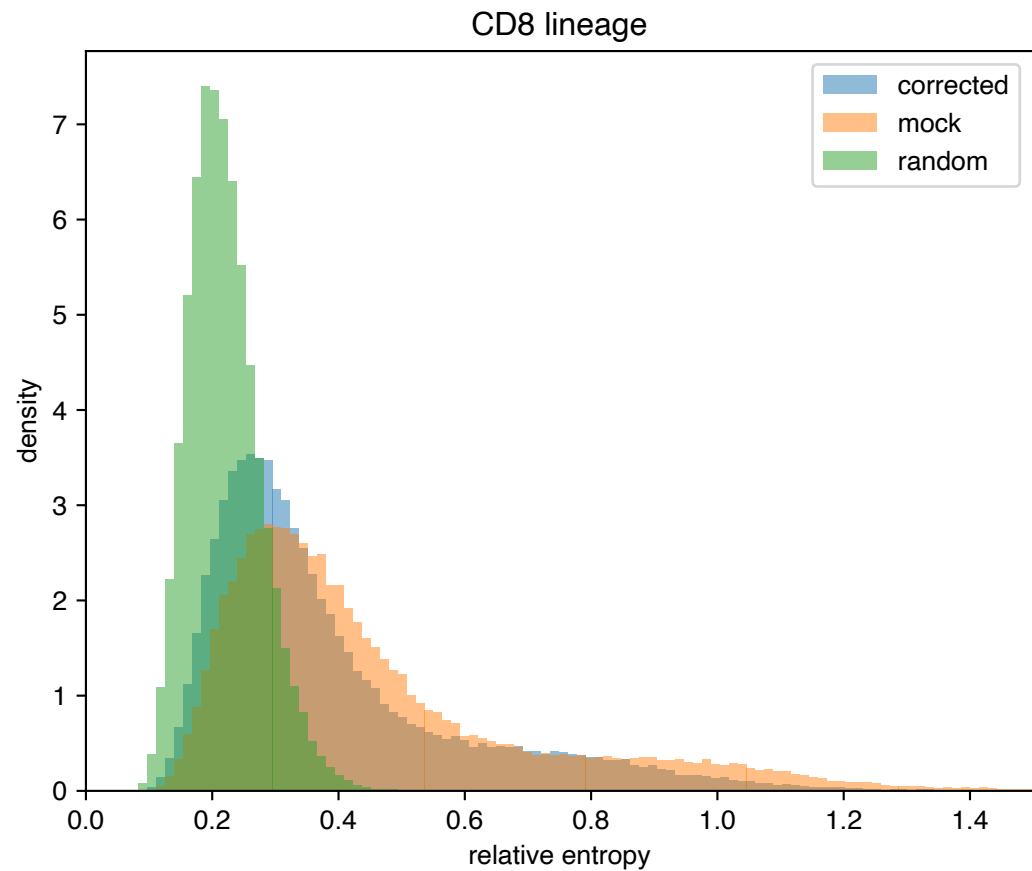

B

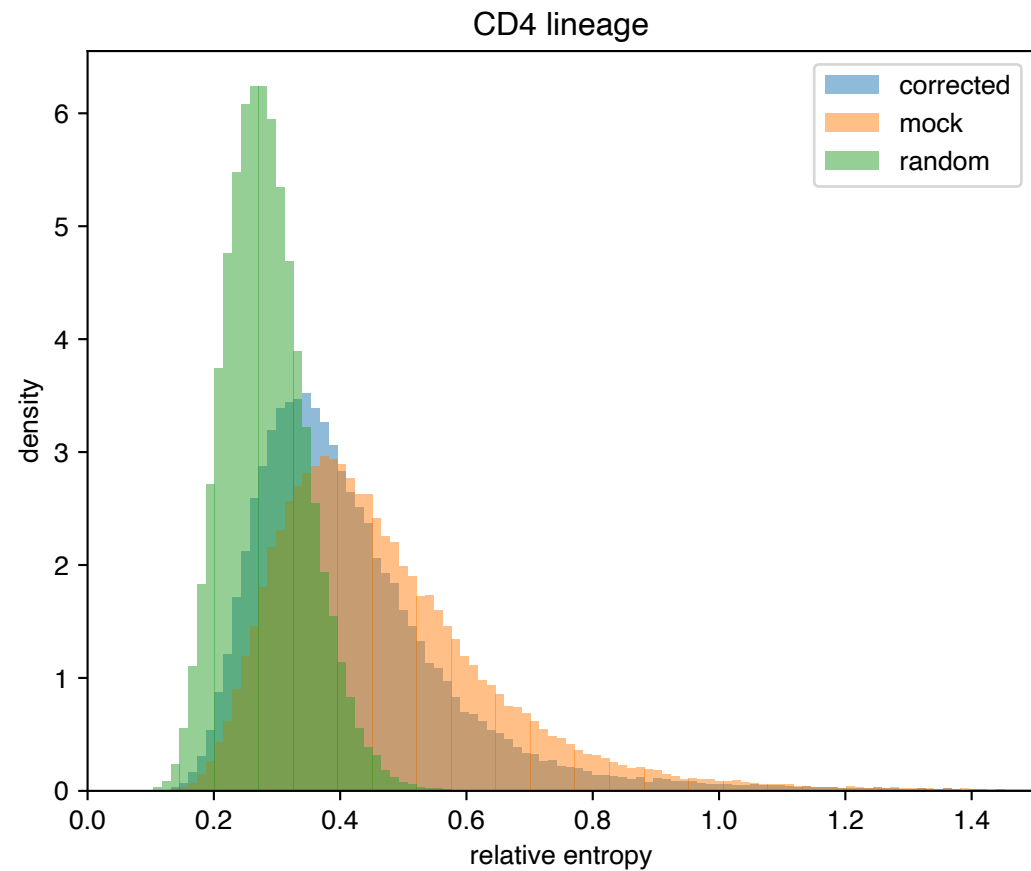

Supplement: S15 Fig — Results are based on data from n = 27 mice. Shown is the entropy of the experimental batch distribution around each cell, using the latent vector z and its nearest neighbors. Values for batch-corrected latent vectors are shown in blue. Mock corrected values are shown in orange, and values for randomized batch information are shown in green. Panels A and B show CD8 and CD4 data, respectively. The distributions are capped at 1.5 as there was a very small number of cells with high relative entropy. (PDF) [file pcbi.1013242.s016.pdf]
