# Supplementary material for: A variational deep-learning approach to modeling memory T cell dynamics
Source: PLoS Comput Biol. 2025 Jul 24;21(7):e1013242. doi: 10.1371/journal.pcbi.1013242 (PMC12360662; doi:10.1371/journal.pcbi.1013242)
Supplement: S4 Fig — Results are based on data from n = 34 mice. A. Number of NP-specific CD8 T cells, as a function of post-infection sampling time. The curve represents a spline fit to the log-transformed T cell counts, and the dashed lines represent the 95% confidence envelope (estimated by bootstrapping residuals). The red curve indicates the number of polyclonal CD8 T cells (cf. Fig 1B). B. The Tet+ fraction of bulk CD8 T cells in the lung niche. Splines are fitted on the logit scale. C. Number of NP-specific CD4 T cells. D. The Tet+ fraction of bulk CD4 T cells. (PDF) [file pcbi.1013242.s005.pdf]

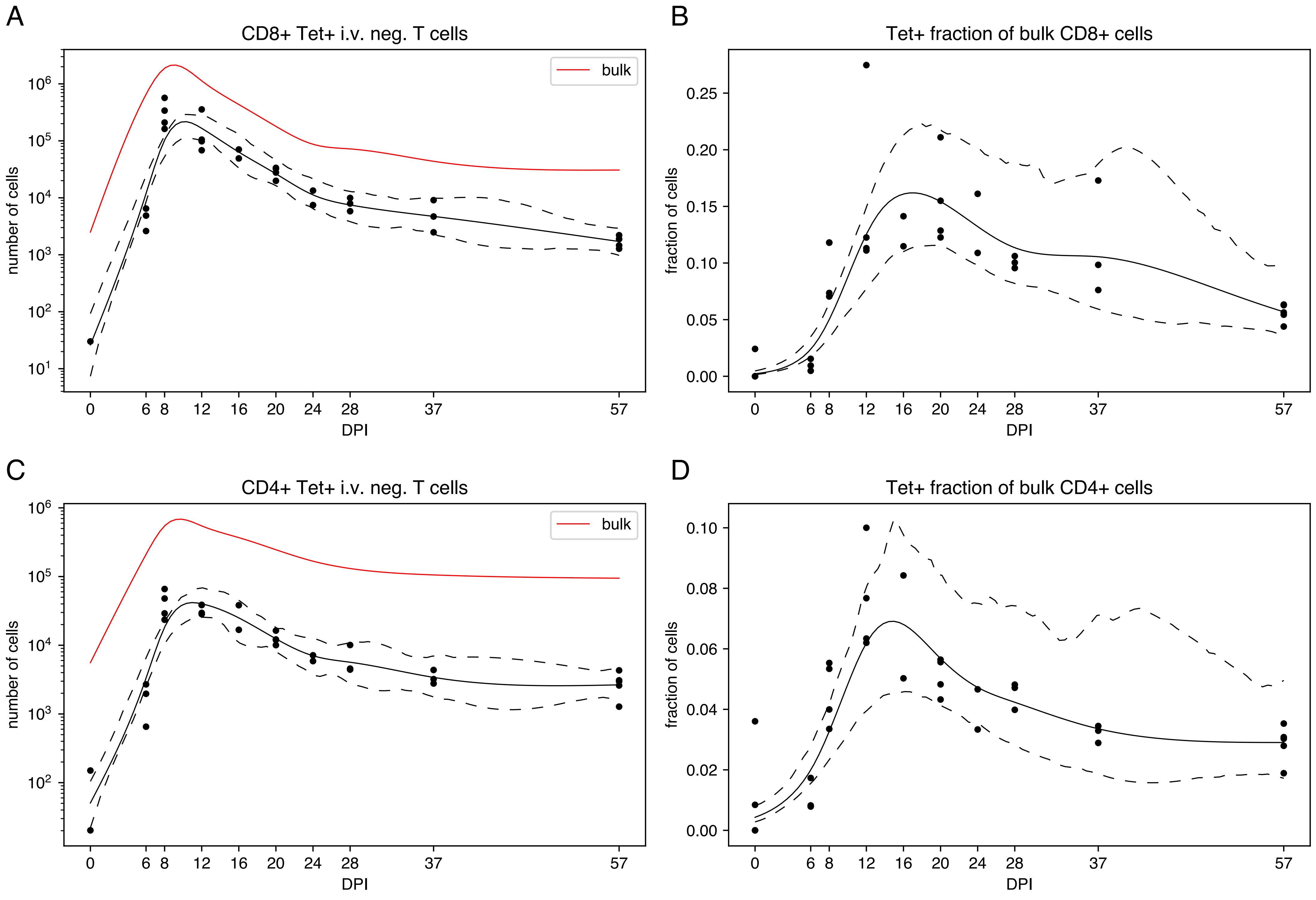

A
CD8+ Tet+ i.v. neg. T cells
bulk
number of cells
DPI
B
Tet+ fraction of bulk CD8+ cells
fraction of cells
DPI
C
CD4+ Tet+ i.v. neg. T cells
bulk
number of cells
DPI
D
Tet+ fraction of bulk CD4+ cells
fraction of cells
DPI
