# Supplementary material for: A variational deep-learning approach to modeling memory T cell dynamics
Source: PLoS Comput Biol. 2025 Jul 24;21(7):e1013242. doi: 10.1371/journal.pcbi.1013242 (PMC12360662; doi:10.1371/journal.pcbi.1013242)
Supplement: Attachment — Submitted filename: rebuttal_PCB_R1.pdf [file pcbi.1013242.s017.pdf]

‘A variational deep-learning approach to modeling memory T cell dynamics’ van Dorp *et al.*

Response to reviewers

We are extremely grateful to both reviewers for their comments – by addressing them, we believe the manuscript has improved substantially. We address all the comments point by point below:

Reviewer #1

In this manuscript, the authors have addressed the challenges of confronting established mathematical modeling techniques with high-dimensional immunological data. The authors have used two different approaches, a sequential and an integrated deep learning-based approach, to understand the heterogeneity during the development and persistence of memory T cells after IAV infection in mice. They have discussed the identifiability issues of model structure and parameters using the sequential approach and used an advanced variational autoencoder model to tackle those issues in the integrated approach. Using the integrated approach, they have found that TRM heterogeneity is maintained long-term by a Bcl-2hi population of cells that can differentiate into functionally distinct subsets. Overall, the ideas presented in the manuscript were interesting and the problems identified are important. Below are some major and minor comments that should be addressed before publication,

Major comments

Since the major conclusions of the paper is primarily based on the integrated approach, I think that the sequential approach part can be condensed down to only 1 figure, rather than 3 figures, with rest of the figures being in the SI. The main figure can just show the clustering part of the flow cytometry data to identify the subsets and 4 model fits to the trajectory data of different subsets.

This is a good idea – the data were being shown multiple times. Figure 2 now shows the essential parts of the sequential approach, and Figure 3 is moved to the SI and covers more technical details. We do think that keeping Figure 1 is important, as it gives a clear overview of the data and might be a good entry point for readers with an experimental background.

The authors have done a good job of describing the issues with the sequential approach. However, I found it difficult to interpret how they have solved these problems of the sequential approach using the integrated approach. For example, in lines 360-370, they talk about the identifiability issues of the sequential approach and how they addressed this in the integrated approach. But, what’s the ultimate conclusion of this part?

Thank you - this is an important point. Identifiability of parameters is an issue for both approaches, and the results from our structural identifiability and sensitivity analyses hold in both cases. However, the practical identifiability analysis was much more feasible to do for the SA than the IA. We do think that the IA suffers from the same limitations in this respect. We now mention this

explicitly in the Discussion (line 589). However, we do point out that the IA provides opportunities to mitigate the issue by making use of distances in the latent space. This appears to work better for the CD8 lineage than the CD4 lineage (Fig). The conclusion is that to fully resolve these issues, we need different kinds of data (line 591), and that the IA could provide a framework to jointly model such data.

Further, the idea and rational behind the Gaussian mixture model used in the integrated approach needs to be explained in detail. Why use this model?

We find a lot of overlap / ambiguity in TRM phenotypes in the SA. Instead of brushing this under the carpet by assigning Leiden clusters, we can accommodate this uncertainty most simply with a GMM. Combining GMMs with dynamical modeling has been proven useful in e.g. epidemiology [1]. GMMs tend to work better in low-dimensional spaces, which we generate here using the VAE.

Other more general clustering methods employ an additional VAE layer to map the latent space to a probabilistic cluster assignment and a cluster-independent latent vector. (This approach is utilized in scANVI). In future work we want to re-examine NN-based clustering, especially when the latent space has a larger dimension (e.g. in the case of scRNAseq or CITE-seq data). For this study, we experimented with this method but found that GMMs with constant variances and time-dependent weights are both conceptually simpler and also prevent the generation of uninformative 'catch-all' clusters that can arise with more general methods.

Also, the authors have mentioned in the manuscript that the generated and predicted cluster frequencies are interdependent in the integrated approach and that can result in better fits. This is somewhat concerning to me even though the authors have tried to validate this approach.

We totally understand your concern. We actually find this feature of the approach very interesting! In many dynamic modeling studies in immunology, this issue does not occur as the processing of the data is completely separate from the model and fitting procedure. In this case we treat the clustering step as a first-class citizen of the model, which leads to this diffusion of data and model predictions that we mention. We wanted to bring attention to this, as it highlights an interesting aspect of integrated modeling. However, this aspect only affects the way we interpret the posterior predictive check (or "model fit") that we present in Fig. 4E. It doesn't invalidate the integrated model, which is essentially fit to the 'immutable', single-cell flow cytometry data. We have rewritten the section discussing this issue (line 375), and expanded our explanation in the Discussion (line 550).

Minor comments

In Fig 1D-G, what are the points and the lines? for each time point, why are there 3-4 points? Do the lines represent a fitted curve already or just mean trends?

Thank you for pointing this out. The lines represent the overall trend, which we inferred using cubic splines. The dashed lines are 95% bootstrap CIs. The explanation of these lines has unfortunately been lost in the editing process. We used the slope of the splines to get rough estimates of the loss

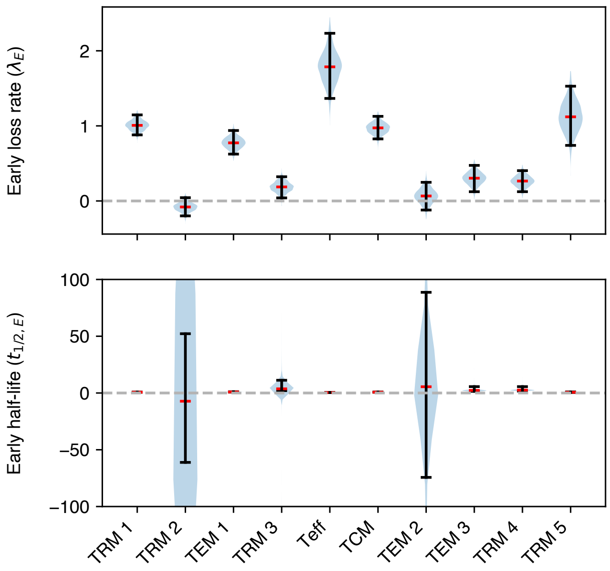

Figure 1: loss rates and half lives. Top panel shows early loss rates for the CD8 lineage estimated with the IA. Bottom panel shows half-lives ($t_{1/2} = \ln 2/\lambda$) where negative half-lives should be interpreted as doubling times.

rates of the T cell populations. We added an explanation to the caption of Figure 1.

Please explain the Leiden clustering method in more detail somewhere in the text or SI. How is this method different from traditional clustering methods? Also, does it result in different clustering patterns for different initial conditions for each run?

In the first part of the Methods section we explain the steps of the sequential approach, but the consensus clustering step was missing. We've added this information (line 660). We also included more details on the Leiden clustering method, which is commonly used in single-cell data analysis.

Indeed, different runs result in different clustering patterns, which is why we added a consensus clustering step to the sequential pipeline (Fig. 2A). In addition to choosing a consensus, we also quantified the variability between runs in terms of entropy (SI Fig 3). This shows which phenotypes are more or less unambiguously defined. This ambiguity was in fact a motivation for using the Gaussian mixture model.

In Fig 3C, Instead of plotting the estimated decay rates, it might be helpful to visualize the estimated half lives from your estimated decay rates?

We agree that it's much more intuitive to present half lives, inter-division times etc. than rates, if possible. What complicates the matter is that we are dealing with self renewing populations so these quantities measure growth and loss at clonal levels. For many of the populations, a zero net loss rate is included in the 95% CrI, corresponding to an infinite half live (or doubling time). For this reason we decided to show rates, and where possible specify half lives in the text to help the reader. We demonstrate the issue in Fig. 1 of this letter. Notice that we had to truncate the

y-axis in the bottom panel to $(-100, 100)$ days. We added a sentence to the Methods to explain this choice (line 694).

Please explain in detail how did you calculate the pattern in fig 5C from fig 5B?

We agree that our current explanation is rather sparse: *"...by sampling from these distributions, we can assign to each cell a single component label"*. To assign a cluster to each cell, we sample from the categorical distribution with probability vector $(p_{n,1}, \dots, p_{n,d})$ (Eqn 7) where d is the number of components / clusters and n is the index of the cell sample. This equation is the direct result of Bayes' rule: The GMM gives the probability density of a latent vector, if we know the true cluster the cell belongs to. We can invert this to get a probability distribution of cluster-membership, given a particular latent vector z.

We now better explain this, and refer to the right equation (caption Fig. 4). We also mention in Methods that we can use $p_{n,i}$ to sample cluster assignments (line 757).

In Fig 5E and 7G, it might be helpful to rename TRM1,2,3 with actual marker labels.

This is a good suggestion. As you can see from figure each subset has its own particular fingerprint of markers. However, we agree that moving back and forth between the model trajectories and the heatmap can be quite difficult, and therefore we have added one or two highly expressed markers to each of the trajectory panels in Figure 4 (CD8) and 6 (CD4), to make it easier for the reader to identify the different subsets. We hope this is a good compromise. We explain this near line 357.

Also what are the dots and the lines represent in these figures? Are the dots data and the lines fitted?

That's right. We've added a clarification in the captions (now Figs 4 and 6).

Reviewer #2

In their paper "A variational deep-learning approach to modeling memory T cell dynamics", van Dorp et al. provide an innovative approach to classify and track phenotypic clusters of resident memory T cells following an influenza infection. Their approach effectively analyzes high-dimensional data generated from low-throughput experiments to gain tractable insights into immunological responses. Through this approach, they offer a critical opportunity to identify and characterize processes underlying the regulation of various immunological responses. Indeed, this method appears applicable to a wide range of biological systems, significantly contributing to the insights gained from mathematical models and measured data. Additionally, this work provides insight into how advances in generative AI can benefit mathematical modeling in life sciences, and may lead to further adoption and development of computational methods.

We very much appreciate your positive assessment!

As I have little knowledge in immunology, I cannot realistically assess the identifiability of cell differentiation reported in their model. However, it is important to point out that identifiability,

especially with the insertion of prior knowledge in the form of modeling constraints, is a major concern in such analyses. The comprehensive analysis and discussion of these issues are, in my view, sufficient and of great use for the careful and considerate application of similar methods that will originate from this approach. Therefore, I only have minor comments:

Line 361: typo: ...learned by by the model...

Thanks for spotting this - corrected

Line 430-432: "We chose to continue with model III as it performed just as well as the full model and enabled us to further explore differentiation pathways" While I agree with this choice, specifically because it performs "just as well", I don't think the reasoning here is sufficiently sound. This also relates to Line 249-251, as there you identify that Model IV can unsurprisingly overfit Model III.

This is a good point. We have added some text at the beginning of the CD4 IA section (line 462). The motivation was also that model III allowed us to explore differentiation without the additional identifiability issues associated with model IV.

Data availability

We have now uploaded the Python scripts, Stan and Pyro models, Jupyter notebooks, and data to Github. The single-cell data files were too large to be hosted on github and have to be downloaded individually from Zonedo (links and instructions are provided on the github repository). Preview of the Zonedo repository is here: `http://bit.ly/3HhMTst`.

References

[1] Boven Mv, Kassteele Jvd, Korndewal MJ, Dorp CHv, Kretzschmar M, Klis Fvd, et al. Infectious reactivation of cytomegalovirus explaining age- and sex-specific patterns of seroprevalence. PLOS Computational Biology. 2017;13(9):e1005719. doi:10.1371/journal.pcbi.1005719.
